# Cross-Hemisphere Study Reveals Geographically Ubiquitous, Plastic-Specific Bacteria Emerging from the Rare and Unexplored Biosphere

**DOI:** 10.1128/mSphere.00851-20

**Published:** 2021-06-09

**Authors:** Brittan S. Scales, Rachel N. Cable, Melissa B. Duhaime, Gunnar Gerdts, Franziska Fischer, Dieter Fischer, Stephanie Mothes, Lisa Hintzki, Lynn Moldaenke, Matthias Ruwe, Jörn Kalinowski, Bernd Kreikemeyer, Maria-Luiza Pedrotti, Gaby Gorsky, Amanda Elineau, Matthias Labrenz, Sonja Oberbeckmann

**Affiliations:** a Leibniz Institute for Baltic Sea Research Warnemuende, Biological Oceanography, Rostock, Germany; b Department of Ecology and Evolutionary Biology, University of Michigan, Ann Arbor, Michigan, USA; c Department of Microbial Ecology, Biologische Anstalt Helgoland, Alfred Wegener Institute Helmholtz Center for Polar and Marine Research, Helgoland, Germany; d Leibniz Institute of Polymer Research Dresden, Dresden, Germany; e Microbial Genomics and Biotechnology, Center for Biotechnology, Bielefeld University, Bielefeld, Germany; f Institute of Medical Microbiology, Virology and Hygiene, University Medicine Rostock, Rostock, Germany; g Sorbonne Université, CNRS, Laboratoire d’Océanographie de Villefranche, UMR 7093 LOV, Villefranche-sur-Mer, France; h Research Federation for the Study of Global Ocean Systems Ecology and Evolution, Paris, France; Clemson University

**Keywords:** *Rhodobacteraceae*, bacterial communities, biofilms, marine microbiology, microplastics, microbiome

## Abstract

While it is now appreciated that the millions of tons of plastic pollution travelling through marine systems carry complex communities of microorganisms, it is still unknown to what extent these biofilm communities are specific to the plastic or selected by the surrounding ecosystem. To address this, we characterized and compared the microbial communities of microplastic particles, nonplastic (natural and wax) particles, and the surrounding waters from three marine ecosystems (the Baltic, Sargasso and Mediterranean seas) using high-throughput 16S rRNA gene sequencing. We found that biofilm communities on microplastic and nonplastic particles were highly similar to one another across this broad geographical range. The similar temperature and salinity profiles of the Sargasso and Mediterranean seas, compared to the Baltic Sea, were reflected in the biofilm communities. We identified plastic-specific operational taxonomic units (OTUs) that were not detected on nonplastic particles or in the surrounding waters. Twenty-six of the plastic-specific OTUs were geographically ubiquitous across all sampled locations. These geographically ubiquitous plastic-specific OTUs were mostly low-abundance members of their biofilm communities and often represented uncultured members of marine ecosystems. These results demonstrate the potential for plastics to be a reservoir of rare and understudied microbes, thus warranting further investigations into the dynamics and role of these microbes in marine ecosystems.

**IMPORTANCE** This study represents one of the largest comparisons of biofilms from environmentally sampled plastic and nonplastic particles from aquatic environments. By including particles sampled through three separate campaigns in the Baltic, Sargasso, and Mediterranean seas, we were able to make cross-geographical comparisons and discovered common taxonomical signatures that define the plastic biofilm. For the first time, we identified plastic-specific bacteria that reoccur across marine regions. Our data reveal that plastics have selective properties that repeatedly enrich for similar bacteria regardless of location, potentially shifting aquatic microbial communities in areas with high levels of plastic pollution. Furthermore, we show that bacterial communities on plastic do not appear to be strongly influenced by polymer type, suggesting that other properties, such as the absorption and/or leaching of chemicals from the surface, are likely to be more important in the selection and enrichment of specific microorganisms.

## INTRODUCTION

Plastic is perhaps the most omnipresent artificial substance on the planet today. An estimated 4.8 to 12.7 million tons of plastic were introduced into marine systems in 2010 alone (1). However, only 70,000 to 270,000 tons, or less than 1%, of marine plastic waste is estimated to be floating at the water’s surface (2, 3). The fate of the remainder of the plastic waste is not known. One hypothesis is that microbial colonization/biofouling leads to accelerated vertical transport of plastic, particularly microplastics, to deeper layers of the oceans (4). In addition to drastically altering the physical and chemical fingerprint of the planet, plastic may have a profound effect on ecosystems by shaping biotic communities.

Every piece of plastic pollution is covered with a complex biofilm community containing a diverse group of eukaryotes, archaea, bacteria, and viruses, though the bacterial component is the most often studied (5–12). Thus, in this paper, the term “biofilm” refers to only the bacterial component. The bacterial communities that inhabit plastic floating at the water’s surface in marine environments are significantly different from the bacterial communities in the surrounding water (5, 8, 9, 11, 13–16). This is not surprising, as bacteria attached to microscopic particles in the water (particle-associated water) are known to differ from those that exist in a free-living state (17, 18).

Currently, it is unknown whether intrinsic qualities of plastic select for specific bacteria that would otherwise be undetectable in marine ecosystems. Controlled incubation experiments have shown that the bacterial communities that form on plastic over short time frames (e.g., days to weeks) are often similar to those found on wood (16, 19). In addition, while some studies have reported that polymer type can distinguish structural differences in biofilms (15, 20), others have found that there are no differences in bacterial biofilm communities between different polymer types (11, 19) or that differences occur only during early biofilm formation (21). However, in low-nutrient environments, measurable distinctions have been found between the biofilm communities on plastic and nonplastic and on different polymer types (16). Of the few studies that have evaluated environmentally sampled plastic across multiple sampling sites, some found that geographical location is more important than the plastic surface in structuring the plastic biofilm communities (13, 22), while others have seen a combination of plastic substrate and geographical drivers (12, 23). There is still a lack of comprehensive comparisons of biofilm communities on plastic particles relative to those on environmentally sampled nonplastic particles. Furthermore, while certain groups of bacteria are found repeatedly in bacterial biofilms, such as *Rhodobacteraceae* and *Sphingomonadaceae*, it is still unclear whether this is due to their high prevalence across all portions of marine ecosystems or because they prefer the plastic biofilm lifestyle (5, 7–9, 11, 12, 14, 16, 19, 20, 24–29). Free-floating plastic pollution does harbor bacteria that are undetectable in the surrounding waters (5, 15, 25), but a comparison to free-floating nonplastic particles is needed to determine whether this is due to the plastic itself or simply its ability to harbor a biofilm.

In this study, we sampled and sequenced the biofilms on microplastic and nonplastic (natural and wax) particles, as well as reference water communities taken from three major water systems across the world: the Baltic, Sargasso, and Mediterranean seas. We addressed the following questions. (i) Is the community structure of environmentally sampled microplastic biofilms more strongly influenced by the plastic properties or environmental conditions? (ii) Are there bacteria that are specific to plastic, i.e., not found on locally sampled nonplastic particles or in the surrounding waters? (iii) Are there reoccurring plastic-specific bacteria found across sampling locations? We expected to find that intrinsic properties of the plastic determine bacterial communities, such that plastic biofilm communities sampled across this broad geographical range would show structural and compositional similarities. Such findings could provide insights into whether certain bacterial taxa constitute a cosmopolitan plastic-specific microbiome in waters around the globe.

## RESULTS

### System characteristics.

**FIG 1:**
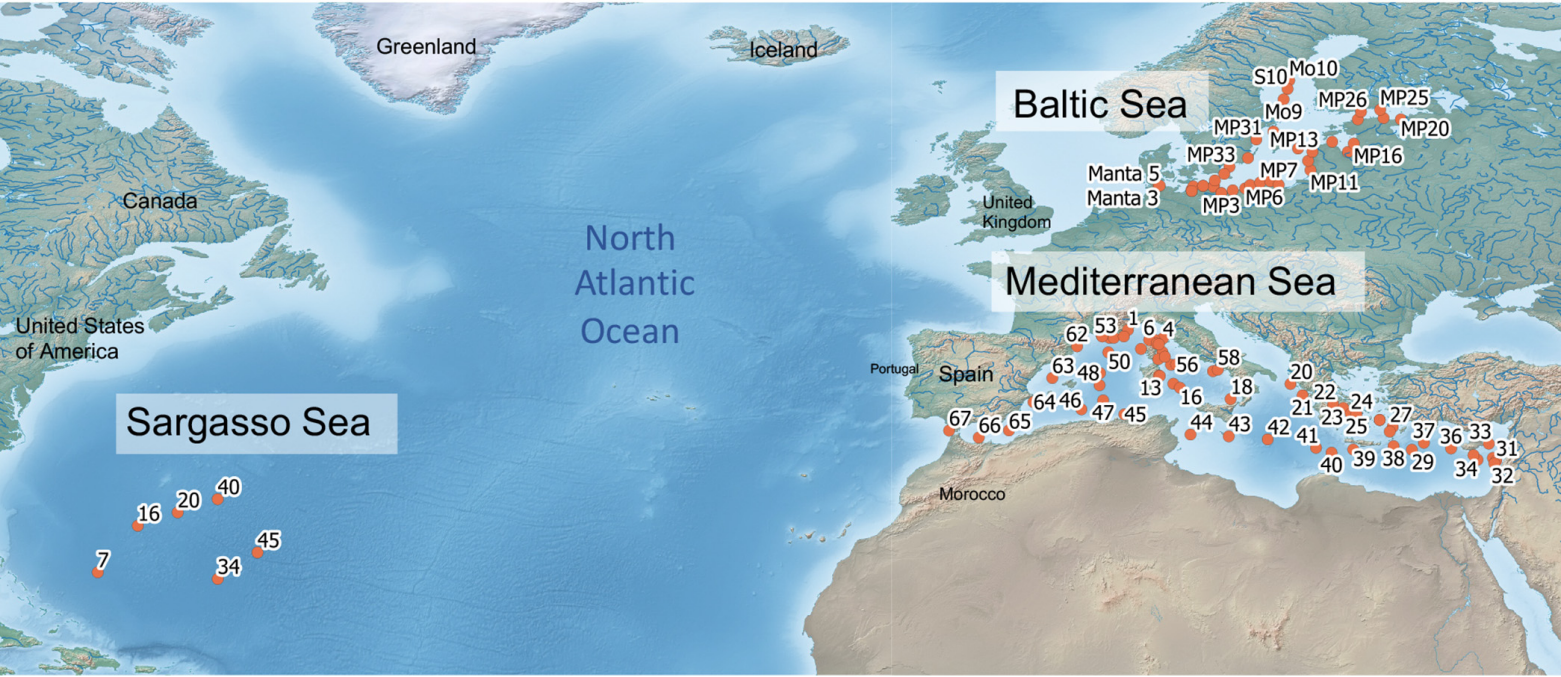
Map of sampling locations. Red dots indicate sampling stations.

Bacterial biofilm communities on sampled microplastic, natural, and wax debris from the Baltic, Sargasso, and Mediterranean seas were compared to the particle-associated and free-living microbial communities from the surrounding water (Fig. 1). Particle-associated (>3 μm) and free-living (3 μm to 0.22 μm) water fractions were analyzed separately throughout this analysis due to their containing two distinct communities of bacteria (17, 18). The average surface temperature and salinity differed among the three environments. The average salinity of the Baltic Sea in the sampling period was 5.58 practical salinity units (PSU) (range, 1.16 to 7.39), while average salinity of the Sargasso was 36.68 PSU (range, 36.58 to 36.75) and that of the Mediterranean Sea was 38.33 PSU (range, 37.7 to 39.51). The temperature of the Baltic Sea during sampling (average, 18.13°C; range, 16.57 to 21.92°C) was lower than that of the Sargasso (average, 23.67°C; range, 21.08 to 25.39°C) and the Mediterranean (average, 28.74°C; 22.65 – 29°C) seas. Across all locations combined, biofilms from 145 microplastic samples, 16 micrononplastic samples, 74 particle-associated (>3-μm filtered) water fraction samples, and 77 free-living (3-μm to 0.22-μm filtered) water fraction samples were evaluated in this study (Tables 1 and 2).

**TABLE 1:** Samples included in the study

Sea	Plastic	Natural	Wax	3-μm water communities	0.22-μm water communities	Total
Baltic	28	15	2	40	47	132
Sargasso	78	1	0	18	14	111
Mediterranean	13	3	0	16	16	48
Total	119	19	2	74	77	

**TABLE 2:** Plastic and particle types included in the study

Sample type^*a*^	No. from sea			Total
	Baltic	Sargasso	Mediterranean	
Plastic				
ABS	1	0	0	1
Hostalen HDPE	0	1	0	1
PP fiber	1	0	0	1
PAAM	0	0	1	1
PE	8	66	7	81
PET	1	0	1	2
PP	9	9	2	20
PS	6	1	1	8
PVC	0	0	1	1
PU	0	1	0	1
TEF	1	0	0	1
Varnish	1	0	0	1
Natural particles				
Cellulose	1	0	0	1
Natural particle	4	0	1	5
Natural particle fiber	9	1	2	12
Protein	1	0	0	1

a ABS, acrylonitrile butadiene styrene; HDPE, high density polyethylene; PP, polypropylene; PAAM, polyacrylamide; PE, polyethylene; PET, polyethylene terephthalate; PS, polystyrene; PVC, polyvinyl chloride; PU, polyurethane; TEF, thermoplastic elastomer fiber.

### Intracommunity (alpha) diversity.

Overall, biofilm communities from both microplastic and nonplastic samples had lower mean richness than either particle-associated (>3 μm) or free-living (3 μm to 0.22 μm) water communities (mean observed richness: plastic particles = 273, natural particles = 175, wax particles = 172, particle associated = 413, free living = 307), and these differences were statistically significantly different, except between plastic particles and natural particles and free-living water samples and all comparisons to wax samples, likely due to the lower number of wax particles (*n* = 2) (Kruskal-Wallis with *post hoc* Dunn’s test, *P* ≤ 0.001) (Fig. S1; Table S1). Though natural biofilm communities had slightly lower richness than plastic biofilm communities, this was not statistically relevant (Fig. S1; Table S1). Since there were only two wax particles in total, comparisons between these communities and the other sample types were not statistically relevant. The evenness of the bacterial communities did not differ statistically between sample types (mean inverse Simpson index: plastic particles = 21.58, natural particles =14.9, wax particles = 13.74, particle-associated water fractions = 26.5, free-living water fractions = 21.1), except between natural particles and particle-associated water (Fig. S1; Table S1) (Kruskal-Wallis with *post hoc* Dunn’s test, *P* < 0.05). Within each location, similar patterns of richness and evenness were observed between sample types as described above (Fig. S2; Table S1).

10.1128/mSphere.00851-20.1 FIG S1 Alpha diversity comparisons between the sample types. Richness (observed number) and evenness (inverse Simpson) were measured between the bacterial communities of the different samples. ***, *P* < 0.001. Statistics are shown in detail in Table S1. Download FIG S1, TIF file, 0.1 MB. Copyright © 2021 Scales et al. 2021 Scales et al. https://creativecommons.org/licenses/by/4.0/ This content is distributed under the terms of the Creative Commons Attribution 4.0 International license.

10.1128/mSphere.00851-20.2 FIG S2 Alpha diversity comparisons between the sample types and across locations. Richness (observed number) and evenness (inverse Simpson) were measured between the bacterial communities of the different sample types and across the different locations. Statistics are shown in detail in Table S1. Download FIG S2, TIF file, 0.1 MB. Copyright © 2021 Scales et al. 2021 Scales et al. https://creativecommons.org/licenses/by/4.0/ This content is distributed under the terms of the Creative Commons Attribution 4.0 International license.

10.1128/mSphere.00851-20.9 TABLE S1 Alpha diversity and beta diversity. For alpha-diversity statistics, means and standard deviations (85) of richness and evenness were compared across sample types and locations using the Kruskal-Wallis test. Statistically significant *P* values are displayed in bold and in red. For beta-diversity statistics, Adonis and betadisper were used to determine whether the sample types and locations had statistically significant differences in centroids and dispersal around centroids, using the Bray-Curtis and Sørensen distance metrices. Statistically significant *P* values are displayed in bold and in red. A dagger indicates that correction for unequal group sizes was applied to the test. Download Table S1, DOCX file, 0.05 MB. Copyright © 2021 Scales et al. 2021 Scales et al. https://creativecommons.org/licenses/by/4.0/ This content is distributed under the terms of the Creative Commons Attribution 4.0 International license.

### Intercommunity (beta) diversity.

**FIG 2:**
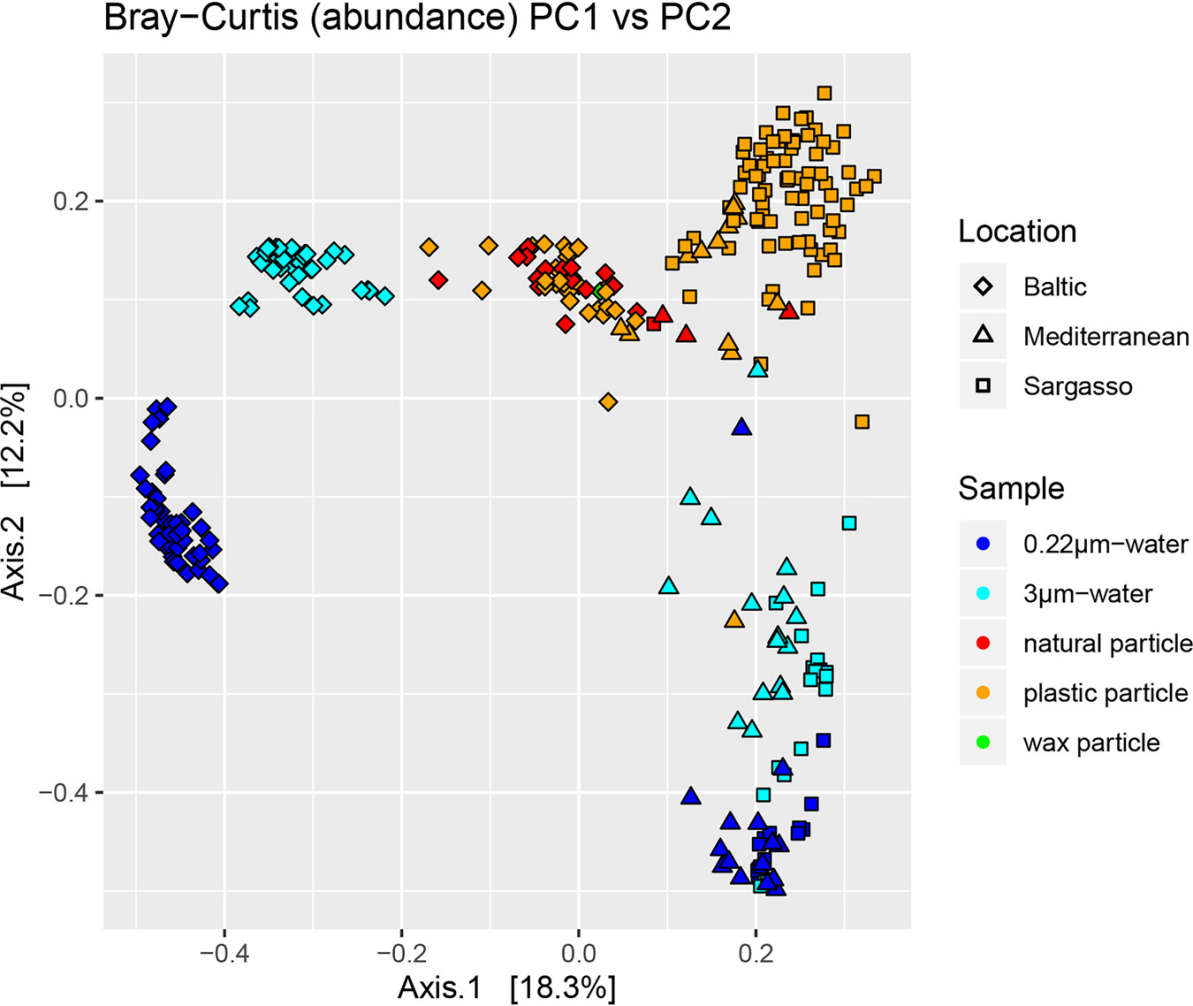
Principal-coordinate analysis (PCoA) of bacterial communities from plastic particles, natural particles, wax particles, particle-associated (>3 μm) water fractions, and free-living (3 μm to 0.22 μm) water fractions. The Bray-Curtis distance metric, which takes into account both abundances and presence/absences of individual OTUs, was used to measure the dissimilarity of each sample. Each symbol refers to a bacterial community. The individual communities are colored based on sample type and have different shapes based on location. More similar communities are closer together in the ordination plot. Results of PERMANOVA and homogeneity of dispersion analysis are found in Table S1.

Biofilm communities from all locations clustered together away from the water communities, suggesting that the type of community (biofilm versus water) is a stronger indicator of final community structure than sample location (Fig. 2; Fig. S3). This was seen when relative abundances of individual operational taxonomic units (OTUs) (Bray-Curtis dissimilarity) (Fig. 2; Table. S1) and when just the presence or absence of OTUs (Sørensen dissimilarity) (Fig. S3; Table S1) were considered in evaluating differences in community structure. Though the centroids between plastic particles from each location were statistically different (Table S1) (permutational multivariate analysis of variance [PERMANOVA], *P* ≤ 0.001), the *R*^2^ values (0.048 to 0.1386) suggest that less than 14% of this difference is explained by the actual communities on the plastic particles being compared. In contrast, comparisons between particle-associated water communities from the different locations returned *R*^2^ values at least twice that of the plastic biofilm communities, and for comparisons between the free-living water communities, *R*^2^ was at least three times that of plastic biofilm communities. Location was still a significant determinant of bacterial community structure across sample types, but more so for the water communities, especially for Baltic Sea water samples (Fig. 2; Fig. S3; Table S1). Water samples from the Sargasso and Mediterranean Sea samples showed little distinction in community structure between the two water fractions (particle associated and free living, 3 μm to 0.22 μm) and between sampling locations. In contrast, the water bacterial communities of the Baltic Sea not only were significantly dissimilar from the Sargasso and Mediterranean Sea water communities but also differed in community structure between the two water fractions (Fig. 2; Fig. S3; Table S1).

10.1128/mSphere.00851-20.3 FIG S3 Principal-coordinate analysis (PCoA) of bacterial communities from plastic particles, natural particles, wax particles, particle-associated (>3 μm) water fractions, and free-living (3 μm to 0.22 μm) water fractions. The Sørensen distance metric, which takes into account just the presence and absence of individual OTUs, was used to measure the dissimilarity of each sample. Each symbol refers to a bacterial community. The individual communities are colored based on sample type and have different shapes based on location. More similar communities are closer together in the ordination plot. Results of PERMANOVA and homogeneity of dispersion analysis are found in Table S1. Download FIG S3, TIF file, 0.2 MB. Copyright © 2021 Scales et al. 2021 Scales et al. https://creativecommons.org/licenses/by/4.0/ This content is distributed under the terms of the Creative Commons Attribution 4.0 International license.

### Influence of plastic type on sampled biofilms.

**FIG 3:**
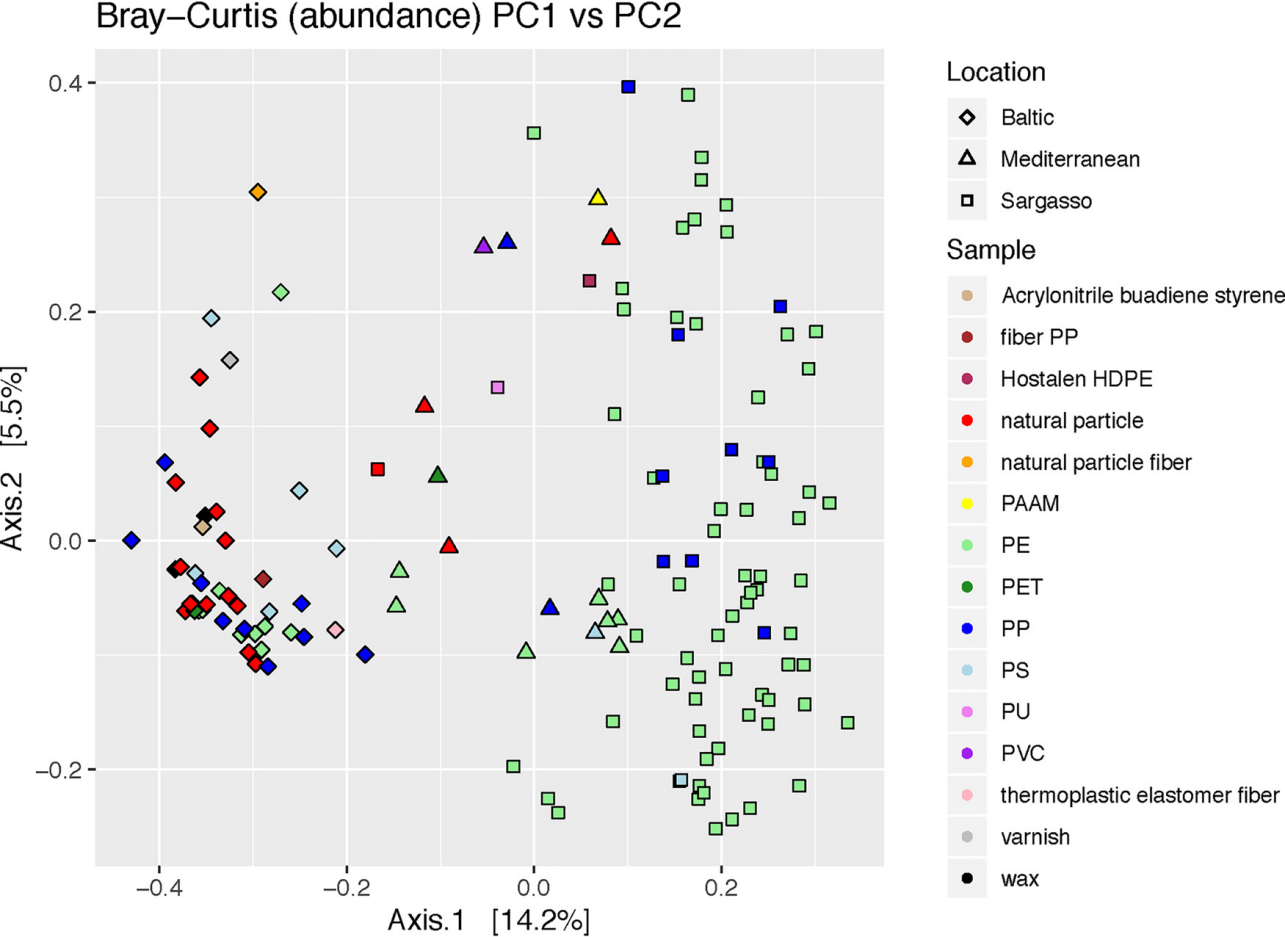
PCoA of bacterial communities on plastic, natural, and wax particles, colored by specific particle type. Bray-Curtis distance metrics were used to measure the dissimilarity of each sample. Each symbol refers to an individual bacterial community. The individual communities are colored based on the type of plastic type and have different shapes based on location. More similar communities are closer together in the ordination plot. Results of PERMANOVA and homogeneity of dispersion analysis are found in Table S1. ABS, acrylonitrile butadiene styrene; HDPE, high-density polyethylene; PP, polypropylene; PAAM, polyacrylamide; PE, polyethylene; PET, polyethylene terephthalate; PS, polystyrene; PVC, polyvinyl chloride; PU, polyurethane; TEF, thermoplastic elastomer fiber.

When all plastic-associated communities were compared, polymer type did not appear to strongly influence the biofilm community structure found of sampled microplastics (Fig. 3). Attenuated total reflectance-Fourier transform infrared (ATR-FTIR) and Raman spectroscopy identification of the sampled microplastics led to 12 polymer categories, with the largest categories being polyethylene (PE) (*n* = 81) and polypropylene (PP) (*n* = 20) (Tables 1 and 2). There was large variance in where the polymer types were found, with the majority of PE (∼82%) and Hostalen GM 6255 high-density PE (HDPE) (*n* = 1) sampled from the Sargasso, all acrylonitrile butadiene styrene (ABS) (*n* = 1), both types of plastic fibers (*n* = 2), and varnish (*n* = 1) sampled from the Baltic, and both polyacrylamide (PAAM) (*n* = 1) and polyvinyl chloride (PVC) (*n* = 1) exclusively sampled from the Mediterranean (Tables 1 and 2). PP represented the polymer with the most even distribution across locations (Baltic, *n* = 9; Sargasso, *n* = 9; and Mediterranean, *n* = 2) (Tables 1 and 2). Thus, while there appeared to be a significant difference between biofilm communities of PE and PP, the two largest plastic types (Fig. 3) (PERMANOVA, *P* ≤ 0.001), this could have been due to the influence of sampling location on polymer type (Table S1). Supporting this observation of low polymer effects, PP-associated communities showed no clustering by polymer type, only by sampling location (Fig. 3).

### Abundant bacteria in biofilm and water communities.

High-abundance OTUs were found across all five bacterial community sample types (plastic particles, natural particles, wax particles, particle-associated water communities, and free-living water communities) (Fig. S4). OTU1, classified as *Alteromonas*, was the most abundant OTU in the plastic biofilm, wax particle, and particle-associated (>3 μm) water communities, as well as among the top 10 most abundant OTUs in the natural-particle biofilms (Fig. S4) and free-living water communities. Of the 20 most abundant OTUs in each group, the plastic biofilm communities shared three OTUs with the natural particle biofilm communities (OTU1_*Alteromonas*, OTU3_*Pseudomonas*, and OTU21_*Pseudoalteromonas*), two OTUs with the wax particle communities (OTU1_*Alteromonas* and OTU3_*Pseudomonas*), four OTUs with the particle-associated water communities (OTU1_*Alteromonas*, OTU3_*Pseudomonas*, OTU6_*Erythrobacter*, and OTU26_*Halomonas*), and one OTU with free-living water communities (OTU1_*Alteromonas*). The sample types that shared the highest number of abundant OTUs were the two water communities (free-living and particle-associated communities), which shared seven OTUs in the top 20. This pattern was also observed on the class level, with similar classes of bacteria found between plastic and nonplastic (natural and wax) biofilms and between the two types of water communities (Fig. S5).

10.1128/mSphere.00851-20.4 FIG S4 Rank abundances of the top 20 OTUs in each sample type. The 20 OTUs with the highest relative abundance in each sample type are displayed by rank from lowest to highest. Download FIG S4, TIF file, 0.7 MB. Copyright © 2021 Scales et al. 2021 Scales et al. https://creativecommons.org/licenses/by/4.0/ This content is distributed under the terms of the Creative Commons Attribution 4.0 International license.

10.1128/mSphere.00851-20.5 FIG S5 Class level of the bacterial families within each sample type and location. Download FIG S5, TIF file, 0.3 MB. Copyright © 2021 Scales et al. 2021 Scales et al. https://creativecommons.org/licenses/by/4.0/ This content is distributed under the terms of the Creative Commons Attribution 4.0 International license.

### Plastic-specific bacteria.

**FIG 4:**
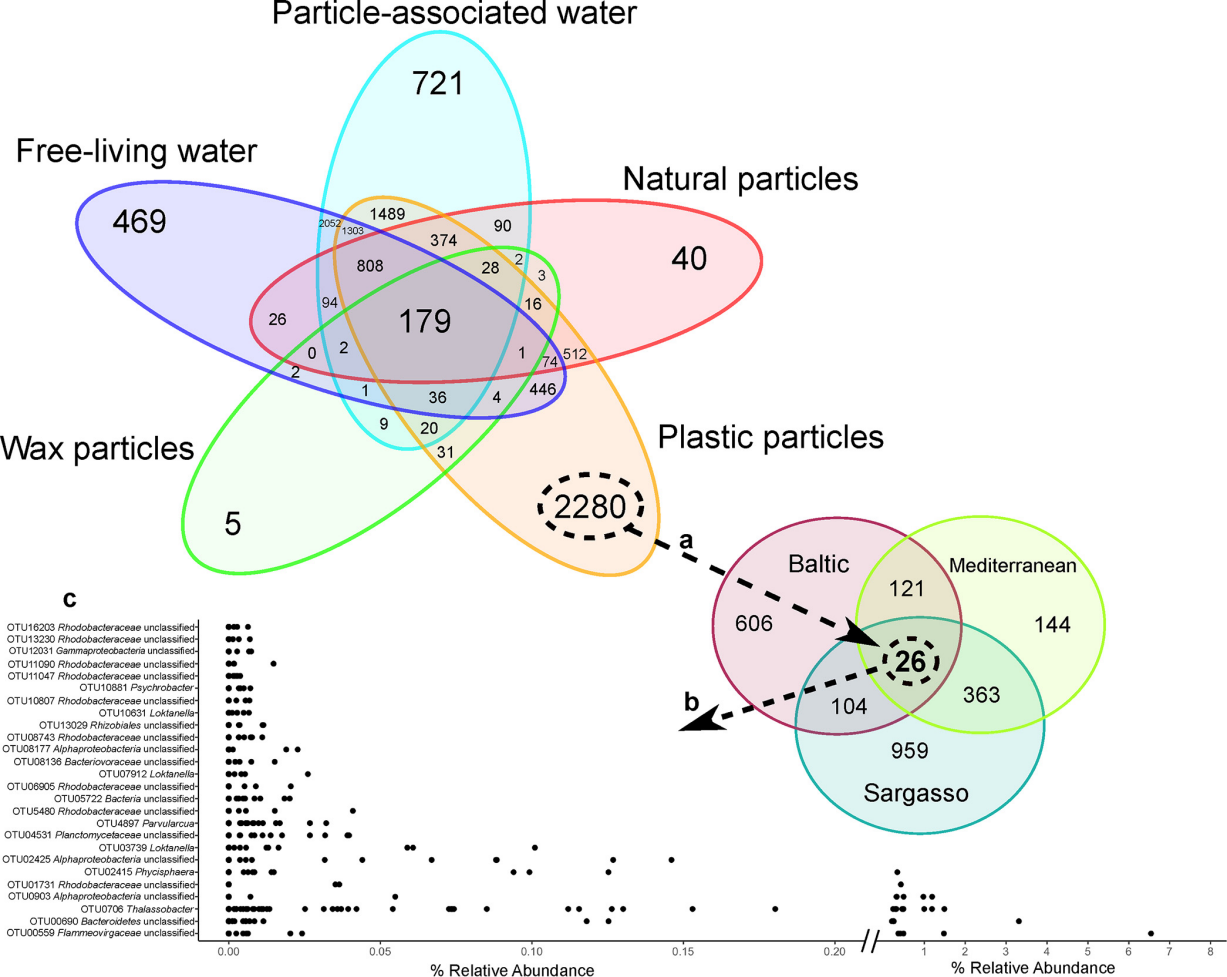
Discovery of plastic-specific OTUs found across all three locations. (a) A total of 2,280 OTUs were present in plastic biofilms but absent from all other sample types in this study. (b) Of the plastic-specific OTUs, 26 were found on samples from all three locations. (c) Relative abundances of the 26 plastic-specific OTUs found across all locations. Each dot refers to a single plastic biofilm community.

Two questions we sought to address were whether plastic-specific taxa could be identified and, if so, whether they reoccurred across habitats. To investigate these questions, we looked for OTUs of plastic biofilm communities that occurred across all locations (Baltic, Sargasso, and Mediterranean seas) but were absent from all other sample types (Fig. 4). A total of 2,280 OTUs were found to be specific to plastic particles, 30% of all plastic-related OTUs (Fig. 4a). Of those, 959 occurred solely in the Sargasso Sea, 144 solely in the Mediterranean Sea, and 606 solely in the Baltic Sea. The Sargasso and Mediterranean plastic communities shared the highest number of plastic-specific OTUs (363) (Fig. 4b). This aligns with the findings of the multidimensional scaling (Fig. 2 and Fig. S3), where the plastic biofilm communities from these two locations showed the most overlap. Of the plastic-specific OTUs that were also specific to each location, differences are seen at the class level: *Actinobacteria* and *Gammaproteobacteria* had higher relative abundances in the Baltic Sea than in the Sargasso and Mediterranean seas, *Verrucomicrobia* were more abundant in the Sargasso Sea, and *Alphaproteobacteria*, *Opitutae*, and TM7 class *incertae sedis* displayed higher relative abundances at the Mediterranean Sea (Fig. S6). Plastic-specific OTUs showed more diversity at the class level (Fig. S6) than OTUs found on plastic but not necessarily specific to it (Fig. S5). In particular, compared to all the OTUs found on plastic from each location (Fig. S5), plastic-specific OTUs had higher levels of unclassified *Proteobacteria*, *Sphingobacteria*, TM7 class *incertae sedis*, and *Verrucomicrobia*, as well as others (Fig. S6).

10.1128/mSphere.00851-20.6 FIG S6 Class level of the plastic-specific, location-specific OTUs. Download FIG S6, TIF file, 0.3 MB. Copyright © 2021 Scales et al. 2021 Scales et al. https://creativecommons.org/licenses/by/4.0/ This content is distributed under the terms of the Creative Commons Attribution 4.0 International license.

Twenty-six plastic-specific OTUs were found on at least one plastic particle from all three locations (Fig. 4b). These OTUs were typically rare (<0.1% relative abundance) members of their communities, except for 6 OTUs whose relative abundances averaged across all plastic samples ranged between 0.16% and 0.85% (Fig. 4c; Table 3). Of these, an unclassified *Flammeovirgaceae* reached relative abundances of 6.55% in the Mediterranean, an unclassified *Bacteroidetes* reached 3.31% in the Baltic Sea, and a *Thalassobacter* reached 1.98% in the Sargasso Sea.

**TABLE 3:** Relative abundances of plastic-specific OTUs across all three locations

OTU	mothur classification, genus level	No of communities	Relative abundance (%)		
			Avg	Minimum	Maximum
OTU00559	Flammeovirgaceae_unclassified	11	0.8504	0.0025	6.5452
OTU00690	Thalassobacter	15	0.2875	0.0011	3.3139
OTU00706	Bacteroidetes_unclassified	42	0.1683	0.0018	1.4915
OTU00903	Alphaproteobacteria_unclassified	7	0.4821	0.0071	1.1920
OTU01731	Alphaproteobacteria_unclassified	3	0.1668	0.0352	0.4288
OTU02415	Rhodobacteraceae_unclassified	10	0.0715	0.0049	0.3399
OTU02425	Phycisphaera	10	0.0609	0.0037	0.1461
OTU03739	Loktanella	9	0.0304	0.0018	0.1010
OTU04531	Planctomycetaceae_unclassified	11	0.0185	0.0036	0.0397
OTU04897	Alphaproteobacteria_unclassified	14	0.0113	0.0037	0.0321
OTU05480	Bacteria_unclassified	4	0.0163	0.0033	0.0409
OTU05722	Rhodobacteraceae_unclassified	9	0.0085	0.0026	0.0201
OTU06905	Alphaproteobacteria_unclassified	3	0.0115	0.0052	0.0205
OTU07912	Bacteriovoracaceae_unclassified	4	0.0094	0.0019	0.0261
OTU08136	Alphaproteobacteria_unclassified	4	0.0071	0.0021	0.0152
OTU08177	Rhodobacteraceae_unclassified	3	0.0144	0.0014	0.0227
OTU08743	Loktanella	6	0.0063	0.0033	0.0111
OTU10329	Rhodobacteraceae_unclassified	4	0.0074	0.0033	0.0114
OTU10631	Rhodobacteraceae_unclassified	5	0.0036	0.0011	0.0067
OTU10807	Rhodobacteraceae_unclassified	3	0.0049	0.0025	0.0068
OTU10881	Loktanella	4	0.0048	0.0033	0.0071
OTU11047	Rhodobacteraceae_unclassified	6	0.0026	0.0015	0.0038
OTU11090	Psychrobacter	3	0.0061	0.0017	0.0148
OTU12031	Gammeoproteobacteria_unclassified	3	0.0056	0.0028	0.0074
OTU13230	Rhodobacteraceae_unclassified	3	0.0039	0.0014	0.0070
OTU16203	Rhodobacteraceae_unclassified	3	0.0036	0.0017	0.0063

**FIG 5:**
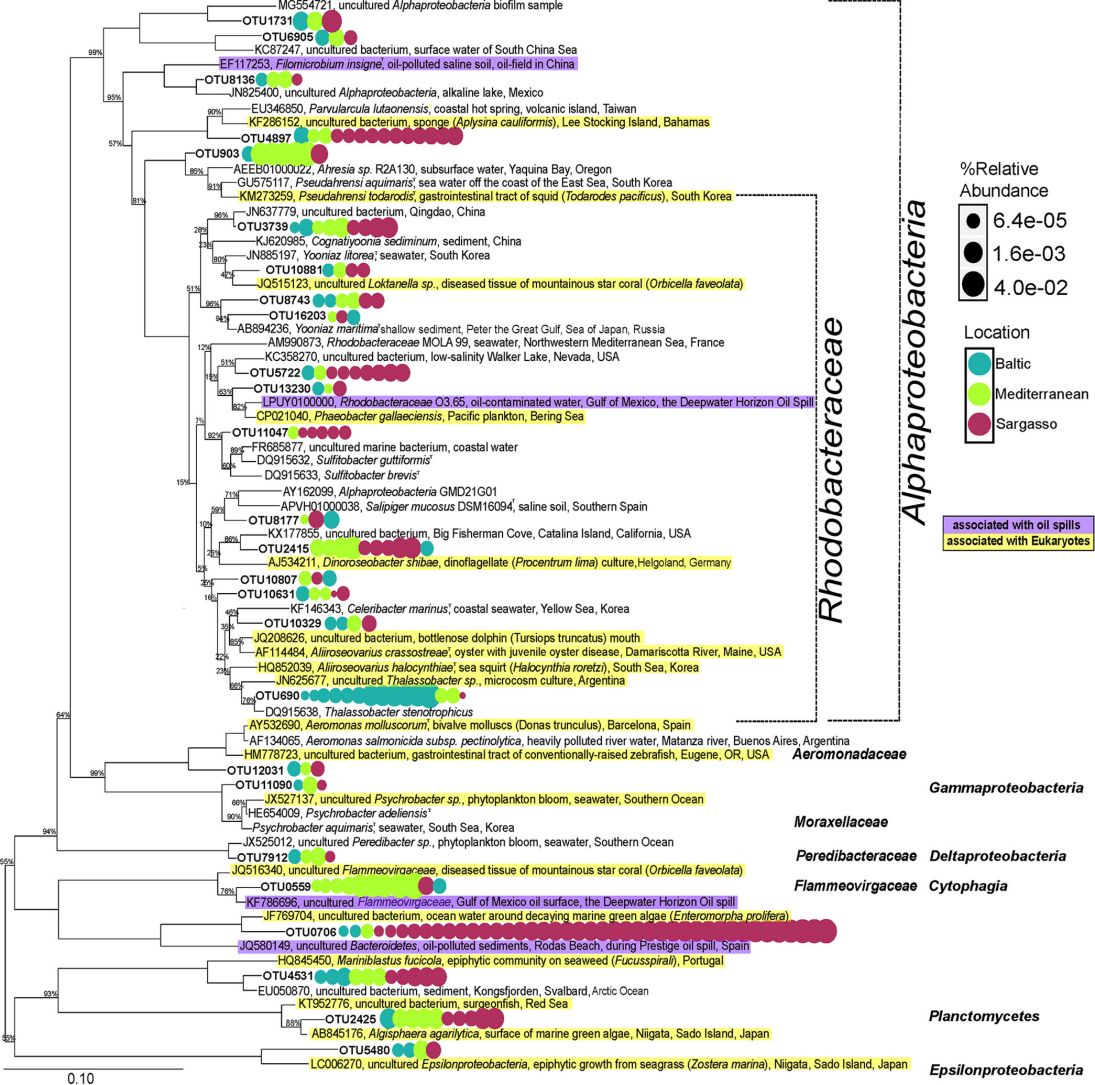
Plastic-specific bacteria and their nearest neighbors. Phylogenetic tree of the 26 plastic-specific OTUs found across all three sampling locations and their nearest neighbors. The plastic biofilms that contain each OTU are depicted by the circles and are colored by location; turquoise indicates that the samples were taken from the Baltic Sea, light green indicates the Mediterranean, and maroon indicates the Sargasso. The size of the dot corresponds to the abundance of that OTU in the sample. Information on the nearest neighbors was obtained from NCBI, and patterns of isolation sources are depicted by highlighting. Nearest neighbors collected from oil spills are highlighted in purple, and those associated with various eukaryotes are in yellow. Neighbor-joining bootstrap tree based on 1,000 iterations, displaying confidence values of >50%. The tree was built in ARB. The bar represents 10 nucleotide substitutions per 100 nucleotides.

We combined three approaches to precisely classify the plastic-specific OTUs found across all three locations: (a) creation of a representative 16S rRNA gene amplicon sequence for each OTU, (b) identification of nearest neighbors via an NCBI BLAST search of each representative sequence, and (c) generation of a phylogenetic tree comprised of the representative sequences (Fig. 5; Table S2). For 25/26 of the OTUs, the most similar previously reported bacterium was represented by an uncultured clone (Fig. 5; Table S2). Two OTUs (OTU5480 and OTU1731) represented novel taxa, with no known sequences detected with at least 97% sequence identity (Table S2). The largest subset of the OTUs were assigned to the family *Rhodobacteraceae* (14/26) (Fig. 5; Table S2). One OTU was classified in each of the families *Aeromonadaceae*, *Moraxellaceae*, *Peredibacteraceae*, and *Flammeovirgaceae*.

10.1128/mSphere.00851-20.10 TABLE S2 Taxonomic identifications of the 26 plastic-specific OTUs that occur across all three locations. BLAST hits of 97% sequence identity or above are highlighted in red. Download Table S2, XLSX file, 0.02 MB. Copyright © 2021 Scales et al. 2021 Scales et al. https://creativecommons.org/licenses/by/4.0/ This content is distributed under the terms of the Creative Commons Attribution 4.0 International license.

To provide ecological context for the plastic-specific OTUs, we determined from which ecosystems their nearest neighbors originated. Four OTUs had nearest neighbors that were first observed in studies describing bacterial populations enriched in response to oil spills (shown in purple in Fig. 5) (30). Of these, OTU559 shared 99.76% identity to an uncultured *Flammeovirgaceae* and OTU13230 shared 98% identity with a cultured and genome-sequenced *Rhodobacteraceae* strain, O3.65 (31) (Table S2). Both of these oil spill-related bacteria were sequenced from oil-contaminated water after the Deepwater Horizon oil spill in the Gulf of Mexico. Furthermore, 16 of the plastic-specific OTUs were most similar to bacteria associated with eukaryotic organisms, including seaweed, seagrass, sponges, squids, coral, plankton, dinoflagellates, algae, oysters, sea squirt, mollusks, zebrafish, surgeonfish, and bottlenose dolphins (shown in teal in Fig. 5).

## DISCUSSION

### Geographically ubiquitous plastic-specific bacteria.

One pressing question within the field of plastic biofilm research is whether plastic-enriched bacteria alter the microbial ecosystem of marine environments by introducing new species or increasing the proportion of otherwise rare species. In our study we found 2,280 OTUs that were specific to plastic biofilms and thus absent or undetectable in the surrounding water and the microbial communities on nonplastic particles. Of these, 26 were found on at least one microplastic particle from each location (Fig. 4b). For the purposes of this study we labeled these 26 plastic-specific OTUs that occur in all three locations as “geographically ubiquitous”; however, it should be noted that some OTUs occur on only one plastic particle per location. Thus, the total number of geographically ubiquitous plastic-specific OTUs could have increased or decreased had more or fewer plastic particles been collected in any or all of the locations. In fact, 30% of all the OTUs found on plastic were unique to plastic (Fig. 4a) and not found in the water communities and or the natural or wax particle biofilms. Taken together, the reoccurring nature of the plastic-specific OTUs across diverse environmental conditions and the large portion of OTUs on plastic being absent on nonplastic particles and in source water suggest an enrichment by properties of plastic, though processes of microbial community assembly other than selection have not yet been evaluated in these habitats (32).

### Geographically ubiquitous plastic-specific bacteria are rare members of their communities.

Interestingly, the majority of the plastic-specific OTUs found at least once in each location occur in low abundance in their corresponding biofilm communities (Fig. 4c). This finding agrees with a previous study that found that the majority (16/23) of plastic-specific OTUs on laboratory-incubated plastic occurred at less than 0.1% relative abundance (33). Collectively, these observations provide evidence that plastic-specific bacteria are part of the “rare biosphere” (33, 34). Furthermore, early plastic colonizers have been identified as coming from the rare biosphere of surrounding water communities (23). While often ignored as being less important due to their low abundances, rare members have more recently been recognized as important contributors to the genetic, metabolic, and functional potential of a community, especially in the degradation of pollutants in marine environments (35–38). As aquatic plastics are known to absorb pollutants from the surrounding water (39), rare members of the plastic biofilm could be important to the biofilm community in the breakdown of these pollutants as they leach from the plastic surface. Rare members of communities are known to provide genetic and functional resilience, allowing communities to adapt to changes in environmental inputs (40), which would be important to the biofilm community of a transient surface-floating plastic particle. More research is needed to understand the function of these low-abundance plastic-specific bacteria; however, their reoccurrence on plastic biofilms across diverse ecosystems suggests their centrality in plastic biofilm communities.

### Indications that properties of plastic may enrich for hydrocarbonoclastic bacteria.

In this study, 15/26 geographically ubiquitous, plastic-specific OTUs were classified into bacterial families associated with hydrocarbon-degrading ability and/or were found to be highly similar to bacteria previously identified in relation to oil spills (Fig. 5; Table S2). Twelve of these were classified as members of the *Rhodobacteraceae*, the family of bacteria most often cited in marine plastic biofilm communities (5, 7–9, 11, 12, 14–16, 19–21, 25–29, 41, 42). In this study, the *Rhodobacteraceae* OTUs that were found to be highly abundant in plastic biofilms were also detected in the surrounding waters, suggesting that these *Rhodobacteraceae* OTUs are found across multiple portions of the aquatic system (Fig. S7). This contrasts with the primarily low-abundance *Rhodobacteraceae* OTUs that were selectively found in plastic biofilms, emphasizing their unique role (Fig. 4). Of the plastic-specific, geographically ubiquitous OTUs, one *Rhodobacteraceae* (OTU13230) and one *Flammeovirgaceae* (OTU559) showed high levels of sequence similarity to bacterial clones identified in studies of the Deepwater Horizon Oil spill (Fig. 5; Table S2) (43, 44).

10.1128/mSphere.00851-20.7 FIG S7 (a) Relative abundances (in percent) of *Rhodobacteraceae* OTUs across all sample types. Each circle represents one OTU. (b) The number of times each of the top 20 most abundant *Rhodobacteraceae* OTUs occurred in each sample type within each location. Download FIG S7, TIF file, 0.3 MB. Copyright © 2021 Scales et al. 2021 Scales et al. https://creativecommons.org/licenses/by/4.0/ This content is distributed under the terms of the Creative Commons Attribution 4.0 International license.

Hydrocarbonoclastic bacteria, like *Rhodobacteraceae* and *Flammeovirgaceae*, are ubiquitous in marine environments, typically existing in low abundance until a massive influx of hydrocarbons through, e.g., an oil spill leads to a rapid shift in local microbial blooms (43). This is not the first study to identify hydrocarbonoclastic bacteria in plastic biofilms (5, 7, 9, 12, 22, 23, 25). Plastic polymers are derived from hydrocarbons, and thus, one hypothesis is that hydrocarbonoclastic bacteria are enriched on plastic due to their ability to utilize the polymer as a carbon source. However, polymer biodegradation by bacteria in marine plastic biofilms is highly unlikely (12). Oceanic plastic is constantly traveling through marine ecosystems, encountering numerous carbon sources more readily available than that of the plastic polymer. One such carbon source is polycyclic aromatic hydrocarbons (PAHs), which plastic readily absorbs from the surrounding waters and concentrates on its surface (45, 46). Plastic entering marine environments also contain numerous additives and organic pollutants that, along with the absorbed hydrocarbons, can leach out of the plastic surface and thereby provide another localized carbon source (47, 48).

Members of the family *Rhodobacteraceae* are known to break down hydrocarbons, such as the PAHs phenanthrene and naphthalene, in environmental settings (49–52). The continuous absorption and leaching of chemicals from the plastic surface could act in nature similar to a traveling, small-scale oil spill, leading to microblooms of hydrocarbonoclastic bacteria as the plastic moves through the marine ecosystem. While some biological-based particles could also potentially absorb and leach chemicals from the surrounding waters, the biofilms on biologically based marine particles do not have the same potential to permanently alter microbial ecology as plastic, since biologically based particles usually biodegrade faster. Plastics do not readily biodegrade and thus have the potential to exist in aquatic systems indefinitely. Additional research is needed to better understand how the chemicals and additives in plastic potentially enrich for the bacteria of plastic biofilms and thus alter the microbial community of marine environments.

### Close relatives of geographically ubiquitous plastic-specific bacteria were sequenced from eukaryotes.

Besides oil spill-dwelling bacteria, many close relatives of plastic-specific OTUs in our study were sampled from eukaryotes. Microplastics are ingested by aquatic organisms and transferred across trophic levels, and through this process, bacteria can be passed from plastic biofilms to the consumer, and vice versa (53–56). This exchange could explain the large number of close relatives of our plastic-specific OTUs that were previously studied in relation to eukaryotes. An additional, but not necessarily independent, hypothesis is that bacteria that prefer biofilm lifestyles are always present at low numbers in marine environments and can be selected for by both larger organisms living in that environment and solid surfaces floating through it. Some of the closest matches to the plastic-specific bacteria were previously identified as phytoplankton-associated microbes (Table S2). This previously reported observation (10, 11, 57, 58) suggests that plastic biofilm-specific bacteria are likely to be those that form interactions with plastic-colonizing eukaryotes.

### Geographically ubiquitous plastic-specific bacteria are understudied.

As is true for many environmentally sampled bacteria, we found that the plastic-specific bacteria that reoccur across habitats represent understudied members of marine communities. The majority of the geographically ubiquitous plastic-specific OTUs were most similar to uncultured bacterial sequences, and some could not be classified past the class level. This means that either these geographically ubiquitous plastic-specific bacteria are difficult to culture or they have not been isolated and sequenced. These findings point to the large potential for microbes of plastic biofilms to possess undiscovered traits and functions. Their promise for biodiversity discovery was recently supported with a “deep-cultivation” approach, in which novel *Planctomycetes*, and even a new phylum, were isolated among others from plastics following an *in situ* experiment in the Baltic Sea (59). Such “diversity-driven” cultivation of bacteria from plastic biofilms is needed to identify the traits that facilitate their existence in this microhabitat, with a focus on the discovery of plastic-specific bacteria and analysis of the unique functions that define their ability to exist in this particular environment (59).

### Plastic biofilm communities show similarities across a diverse range of environments, regardless of polymer type.

In accordance with the hypothesis that it is the properties of plastic, such as the absorption and leaching of chemicals, rather than the surface itself that enrich for certain members of marine ecosystems, we found that plastic biofilm communities were similar to one another across all marine environments sampled regardless of the polymer type. The Baltic, Sargasso, and Mediterranean seas vary with respect to numerous environmental variables, such as temperature, salinity, wind speeds, river inputs, and anthropogenic influences. However, despite the influence of these different environmental factors on microbial community assembly and the large geographical distances between the three seas, the biofilm communities showed similarity across locations (Bray-Curtis dissimilarity) (Fig. 2; Table S1). Sampling location still influenced microbial community structure, as evidenced in the higher similarity, and therefore clustering, between the biofilm and water communities from the Sargasso and Mediterranean seas, bodies of water with more similar temperature and salinity, compared to the Baltic Sea. Furthermore, biofilm communities within each location were more similar to one another when abundances were included in the analysis (Bray-Curtis dissimilarity) (Fig. 2; Table S1) and less so when just presence or absence of OTUs was considered (Sørensen dissimilarity) (Fig. S3; Table S1). These trends suggest that the marine province (Baltic, Sargasso, or Mediterranean) had a stronger influence in selecting which biofilm microbes were present, while the local environment (plastic, natural, wax, particle-associated water, or free-living water community) had a stronger influence on the relative abundances of the microbes present. However, polymer type did not have a strong influence on community structure (Fig. 3). This is best illustrated by the bacterial communities on polypropylene (PP), the plastic type that occurred most evenly across all locations (Tables 1 and 2). While PP-specific similarities in community structure were not identified, PP biofilms were influenced by sampling location (Tables 1 and 2; Fig. 3). These results agree with previous reports that found no relationship between bacterial community structure and polymer type (13, 22). To better understand the niche-specific properties that drive bacterial community assembly on plastics, future work should include additional descriptions of plastic properties beyond just polymer type, such as absorbed pollutants and PAHs.

### Summary.

Microbial communities found on plastic sampled from the Baltic, Sargasso, and Mediterranean seas showed statistically relevant similarities in membership and abundance, while the polymer type of the plastic had no measurable effect on community selection. Plastic-specific OTUs were found that were ubiquitous across all three sampling areas, revealing that plastic repeatedly enriches certain bacteria from the surrounding waters regardless of the particular location. That a large portion of these geographically ubiquitous plastic-specific OTUs were assigned to the family *Rhodobacteraceae* and/or were highly similar to bacteria from previously published oil-spill studies points to a potential future line of research as to which properties of plastic are involved in this enrichment process. We proposed that the ability of plastics to absorb and leach hydrocarbons causes them to act similarly to a travelling, miniature oil spill, selecting for bacteria that can utilize these substances as a carbon source. The discovery of plastic-specific bacteria, low in abundance yet enriched compared to nonplastic and surrounding water samples, points to the potential of plastic pollution to shift aquatic microbial communities, such as in plastic hot spots. Furthermore, the large number of geographically ubiquitous plastic-specific OTUs in this study that were highly similar to uncultured or unclassified bacteria highlights the enormous potential of plastic to harbor undiscovered bacteria with unique traits and functions. More research is needed into the selection processes and community dynamics that influence the fate of plastic-specific bacteria on the omnipresent plastic pollution in aquatic systems.

## MATERIALS AND METHODS

### Sampling.

Microplastic was sampled with a manta trawl with a 300-μm mesh net from the surface waters of the Baltic and the Sargasso seas and with a 333-μm net mesh from the Mediterranean Sea (Fig. 1). The sampling was part of several large sampling campaigns, including cruise POS488 in the Baltic Sea, cruise MSM41 in the Sargasso Sea, and the TARA Mediterranean expedition. The Baltic Sea is bordered by nine European countries, with ∼8,000 km (5,000 miles) of shore. Due to the input of many rivers and other fresh bodies of water, the salinity of the Baltic Sea is highly variable across its transect (e.g., 2.6 to 30.9 PSU in 2008) (60) and on average is lower than that of either the Sargasso or Mediterranean Sea (average for this study, 5.58 PSU). The Baltic also experiences year-round high winds and has water temperatures lower than the other two bodies of water (annual range of ∼0 to 17°C on average, between 1990 and 2018) (61). The Sargasso Sea is in the North Atlantic Ocean, off the east coast of North America, and has no actual coastline, no direct inputs from the land, famously little wind to no wind over the water’s surface, higher salinity (average for this study, 36.68 PSU), and year-long high temperatures (average for this study, 23.65°C) (62). The Mediterranean Sea is located between Africa and Europe, is bordered by 21 countries, and has ∼46,000 km of coastline, variable winds (63), and the highest water temperatures of the three (average for this study, 28.16°C) and the highest salinity (average for this study, 38.33 PSU).

From each location, 1-liter seawater samples were concentrated via serial filtration, first through a 3-μm filter (Isopore membrane filter; Sigma; TSTP04700) to capture the particle-attached microbial community and second through a 0.22-μm filter (Millipore Express Plus membrane filter; Millipore Sigma; GPWP04700) to capture the free-living microbial community. Manta trawl contents were collected in the net cod ends and rinsed with sterile-filtered seawater. Particles and filters were transferred to empty Eppendorf cups (Baltic and Sargasso) or cryo-safe vials containing 1 ml of RNAlater (Thermo Fisher Scientific; AM7020) (Mediterranean). Filters and sampled microplastic were flash-frozen and stored at −80°C until further analysis. Baltic Sea samples were taken between 22 August 2015 and 15 May 2018, Sargasso Sea samples were taken between 5 April 2015 and 24 April 2015, and Mediterranean samples were taken between 1 June 2014 and 8 November 2014.

During the sampling campaigns, salinity and temperature were measured across all three environments. During the Baltic and Sargasso Sea sampling campaigns, temperature and salinity were determined using a conductivity-temperature-depth probe mounted on a rosette sampler. For the Mediterranean Sea sample campaign, temperature and salinity were determined using a SeaBird SBE45 probe.

### Identification and quantification of microplastics using ATR-FTIR and Raman spectroscopy.

Collected particles were taken back to the laboratory, and polymer identification was carried out initially as described by Lorenz et al. (64). All putative plastic particles were identified individually using an ATR-FTIR unit (Bruker Optik GmbH). The IR spectra were collected in the spectral range of 400 to 4,000 cm^−1^ and compared against a reference library (65). Particles with a match of at least 700 (of 1,000) were counted as safely identified. If the match ranged between 600 and 700, the spectra were manually compared to database spectra and evaluated based on expert knowledge, as suggested by other studies (66, 67). During this process some of the particles were identified as being natural particles but were kept in the analysis as a comparison to the plastic particles. In addition, a subset of particles were unable to be fully identified via ATR-FTIR, likely due to their small size. These particles were selected for further analysis by Raman spectroscopy. Particles large enough for manual handling were analyzed via single point measurements using a WITec alpha 300R Raman microscope (laser wavelength, 532 nm; grating, 600 lines/mm; integration time, 0.5 s; coadded spectra, 20; spectral range, 150 to 3,600 cm^−1^). For convenient Raman analysis, the contents of each tube were filtered onto a silicon filter with a 10-μm pore size, as described previously (68). The filters were analyzed with an automated combination of optical particle analysis and Raman microspectroscopy using GEPARD software (68). Optical imaging was performed with a 20× objective in dark-field mode. Raman measurement parameters were chosen as described above, except for coadding 5 instead of 20 spectra. The Eppendorf tubes holding the samples were made of polypropylene, so to monitor the sample contamination from the tubes, 15 blank samples were measured. Since in the blank samples the PP particle count stayed below 200, a fragmented particle was identified as PP if the PP particle count of a sample exceeded this value. Spectral analysis via database search was performed with TrueMatch software (WITec) and in-house-curated databases. For the single point measurements of the larger particles, all results of the automated database search were evaluated by an experienced spectroscopist. For the GEPARD-based analysis of the filtrated samples, database search results with a hit quality index (HQI) of <5 (Pearson correlation coefficient) were discarded, and the remaining results were also checked by an experienced spectroscopist.

Spectrum analysis revealed that two additional particles were natural in origin and two other particles consisted of wax. As wax can be made of natural (beeswax) or artificial (paraffin) components, we decided to keep wax as a separate category outside natural and plastic particles.

In the end, 21 particles could not be sufficiently identified by ATR-FTIR and Raman spectroscopy and were further excluded from additional analysis.

### DNA isolation.

The total DNA from each collected particle and water filter from the Baltic and Sargasso seas was isolated using a modified protocol developed previously (69). Briefly, 700 μl Tris/saline/EDTA buffer and 19 μl lysozyme (10 mg/ml) were added to the microcentrifuge tube containing one particle or one filter and incubated at 37°C for 1 h. Next, 74 μl Tris-EDTA and 44 μl SDS-Tris-EDTA were added to each tube and incubated at 50°C for 60 min. Each tube was centrifuged at 8,000 × *g* for 10 min, and the supernatant was transferred to a new sterile Eppendorf tube, leaving the particle or filter in the old tube. Next, 1/10 volume of NaCl (5 M) and 1 volume of phenol-chloroform (1:1) was added and centrifuged at 8,000 × *g* for 10 min. The supernatant was transferred to a new Eppendorf tube, an equal volume of −20°C isopropanol was added, and the tube was left in the freezer overnight. The next day, the tube was centrifuged at 10,000 × *g* at 4°C for 20 min. The supernatant was discarded, and the pellet was washed with 500 μl 75% ethanol (EtOH). This step was repeated twice, for a total of three times. At the end of the washing, the pellet was dried and then resuspended in 50 μl PCR-grade water. The yield of isolated DNA was measured with a NanoDrop spectrophotometer. Empty microcentrifuge tubes were included as blank controls.

Total DNA from Mediterranean Sea samples was extracted using a modified Qiagen DNeasy blood and tissue extraction kit (Qiagen; catalog no. 69506), which includes additional steps of rinsing each filter or plastic piece in sterile 1× phosphate-buffered saline (PBS) prior to cell lysis and homogenizing the lysate with a QIAshredder column (Qiagen; catalog no. 79656) prior to DNA capture. DNA was eluted in 50 μl of buffer AE (https://www.protocols.io/view/water-sampling-onto-filters-for-nucleic-acids-sequ-jegcjbw).

Laboratory controls, in the form of microcentrifuge tubes containing only the extraction reagents without sample material, were processed alongside the experimental samples. Four controls were processed with the Baltic Sea samples, two with the Sargasso Sea samples, and two with the Mediterranean Sea samples.

### PCR and sequencing.

Isolated DNA was diluted with PCR-grade water to the average lowest DNA in the data set (3 to 10 ng/μl). Amplification of the V3 and V4 variable regions of the 16S rRNA gene was performed with primers modified from reference 70: Pro341-XT, 5′-TCGTCGGCAGCGTCAGATGTGCAGCCTACGGGNBGCASCAG-3′, and Pro805-XT, 5′-GTCTCGTGGGCTCGGAGATGTCTACNVGGGTATCTAATCC-3′, with Kapa HiFi HS RM (Roche; catalog no. 07958935001) as the polymerase. The PCR protocol consisted of 3 min of denaturation at 95°C, followed by 25 cycles of 95°C for 30 s, 55°C for 30 s, and 72°C for 30 s and a final extension at 72°C for 5 min. Amplified DNA was stored at 4°C until further processing. Successful amplification was confirmed by agarose gel electrophoresis (1.2% [wt/vol] in 1× Tris-borate-EDTA [TBE] buffer at 75 V) and imaged in a ChemiDoc MP imaging system (Bio-Rad). The resulting amplicons had sizes of roughly 450 bp. All further steps in library preparation were performed according to the Illumina 16S protocol Metagenomic Sequencing Library Preparation. Briefly, PCR cleanup, index PCR, PCR cleanup 2, library quantification, normalization and pooling were performed according to the above-referenced manual. Bioanalyzer DNA 1000 chips (Agilent Technologies) and Qubit kits (Thermo Fischer Scientific) were used for quantity and quality controls of each individual sample library and the final library pool. A 10% PhiX control was spiked into the final pool. Four picomoles of the final library pool was subjected to one 50-cycle V2 chemistry test run in order to check equal distribution of reads across all individual libraries. This was followed by a main sequencing run using a 600-cycle V3 chemistry kit on an Illumina MiSeq machine. During the run, roughly 1,000 (raw density, K/mm^2^) clusters were sequenced, generating ca. 15 million reads passing filter specifications. Over 75% of the sequencing and index reads were found with a Qscore of ≥30. All raw data fastq files were recovered from the machine and used for further sequence data processing as outlined below.

### Sequence data processing.

The Mothur standard operating procedure (SOP) was followed for processing of raw sequences into operational taxonomic units (OTUs) (https://mothur.org/wiki/miseq_sop/) with the following parameters: permitted sequence length = 420 to 480 bp; maximum number of ambiguous bases per sequence = 0; maximum number of homopolymers per sequence = 8; taxonomy assignment with Wang classification and the SSSURef_123_SILVA database (required a bootstrap value of ≥80%), and operational taxonomic units at 97% (71). The taxonomy and OTU tables produced were input into R (v 3.6.2)/Rstudio (v 1.1.383) (72) and used to create phyloseq objects (73) for all downstream analysis. Chloroplasts, mitochondria, eukaryotes, and unknown sequences, OTUs with a total abundance of ≤2, and samples with fewer than 10,000 reads were removed. In this process, all laboratory controls but one were removed from the analysis (Fig. S8).

10.1128/mSphere.00851-20.8 FIG S8 (a) Rank abundances of the top 20 OTUs in the laboratory control sample, displayed as number of sequences. Mothur taxonomic identifications are displayed. (b) Principal-coordinate analysis (PCoA) of bacterial communities from plastic particles, natural particles, wax particles, particle-associated (>3 μm) water fractions, and free-living (3 μm to 0.22 μm) water fractions. The Bray-Curtis distance metric, which takes into account both abundances and presence/absences of individual OTUs, was used to measure the dissimilarity of each sample. Each symbol refers to a bacterial community. The individual communities are colored based on location and have different shapes based on sample type. Download FIG S8, TIF file, 0.3 MB. Copyright © 2021 Scales et al. 2021 Scales et al. https://creativecommons.org/licenses/by/4.0/ This content is distributed under the terms of the Creative Commons Attribution 4.0 International license.

### Alpha and beta diversity and statistical analysis.

All diversity and statistical analyses were carried out with the vegan package (74). Species richness (75) and evenness (76) were calculated based on a subsample of the minimum number of reads (10,502). Kruskal-Wallis and Kruskal-Dunn tests with chi-square *post hoc* tests were used to test the significance in richness and evenness between the sample types (plastic, nonplastic, particle-associated water communities, and free-living water communities) between the locations (Baltic, Sargasso, and Mediterranean seas) and the sample types within each location (Table S1).

To elucidate community differences between plastic biofilms, nonplastic biofilms, particle-associated water communities, and free-living water bacterial communities across the Baltic, Sargasso, and Mediterranean seas, we utilized two dissimilarity metrics. The Bray-Curtis dissimilarity metric takes into account both relative abundances of individual OTUs and the presence and absence of each OTUs (77), while the Sørensen dissimilarity metric takes into account just the presence or absence of the individual OTUs (78). Furthermore, we looked at beta-diversity through the lens of both species composition and overall community structure (79). To determine whether communities are different in species composition across the habitats, we measured the difference in centroids with PERMANOVA (80), and to compare structural community differences, we measured homogeneity of dispersion (79). *P* values were obtained based on 999 permutations. To visualize the patterns of similarities between communities, principal-coordinate analysis (PCoA) was performed (81).

### Taxonomic identification of plastic-specific OTUs.

The representative sequences for the geographically ubiquitous plastic-specific OTUs were obtained with the get.oturep call from mothur (https://mothur.org/wiki/get.oturep/). These representative sequences were used to perform BLASTn searches against the entire nonredundant nucleotide collection of NCBI, optimizing for highly similar sequences (Megablast) (82). These OTU representative sequences were also used to create a neighbor-joining bootstrap tree based on 1,000 iterations using the ARB Silva file SSU_138_Ref_NR_99 (83). The sequence ID for each close neighbor in the phylogenetic tree was used to obtain additional metadata from NCBI for that sequence (84).

### Data availability.

All raw sequence files, including sequencing controls, are available from the NCBI Short Read Archive (SRA) database (BioProject no. PRJNA632000).
