# Supplementary figures and images for: Cross-Hemisphere Study Reveals Geographically Ubiquitous, Plastic-Specific Bacteria Emerging from the Rare and Unexplored Biosphere
Source: mSphere. 2021 Jun 9;6(3):e00851-20. doi: 10.1128/mSphere.00851-20 (PMC8265672; doi:10.1128/mSphere.00851-20)

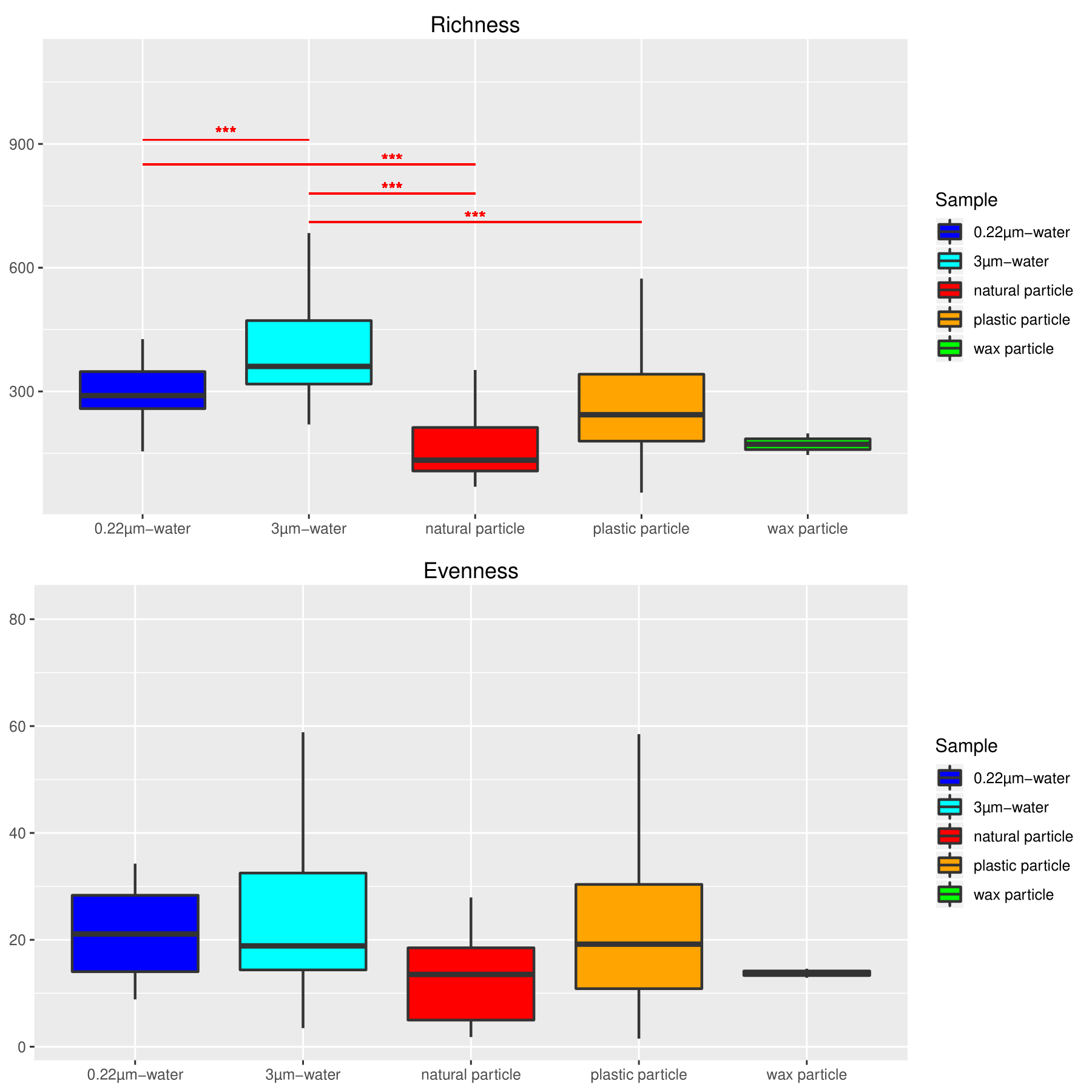

Supplement: FIG S1 [file msphere.00851-20-sf001.tif]

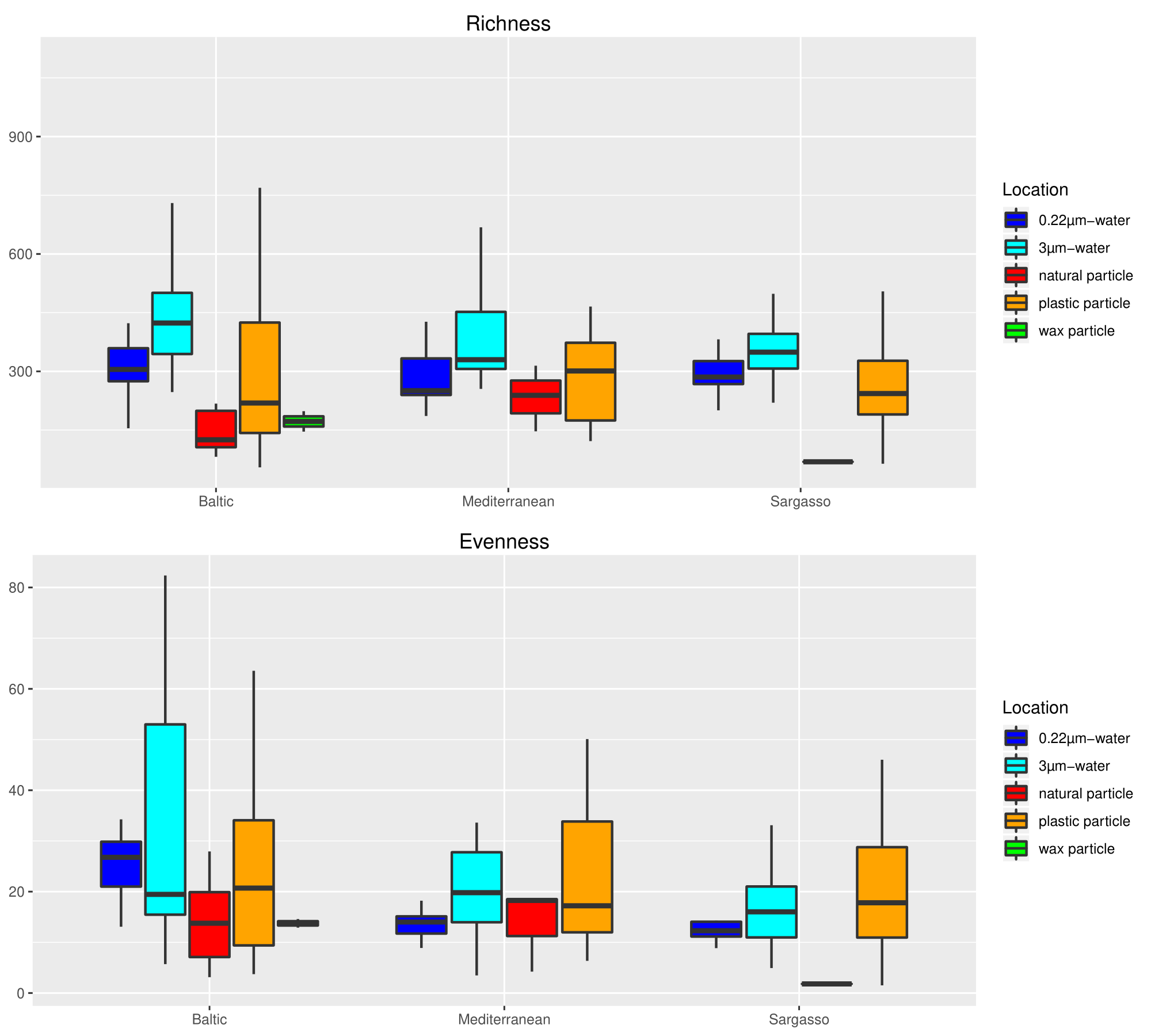

Supplement: FIG S2 [file msphere.00851-20-sf002.tif]

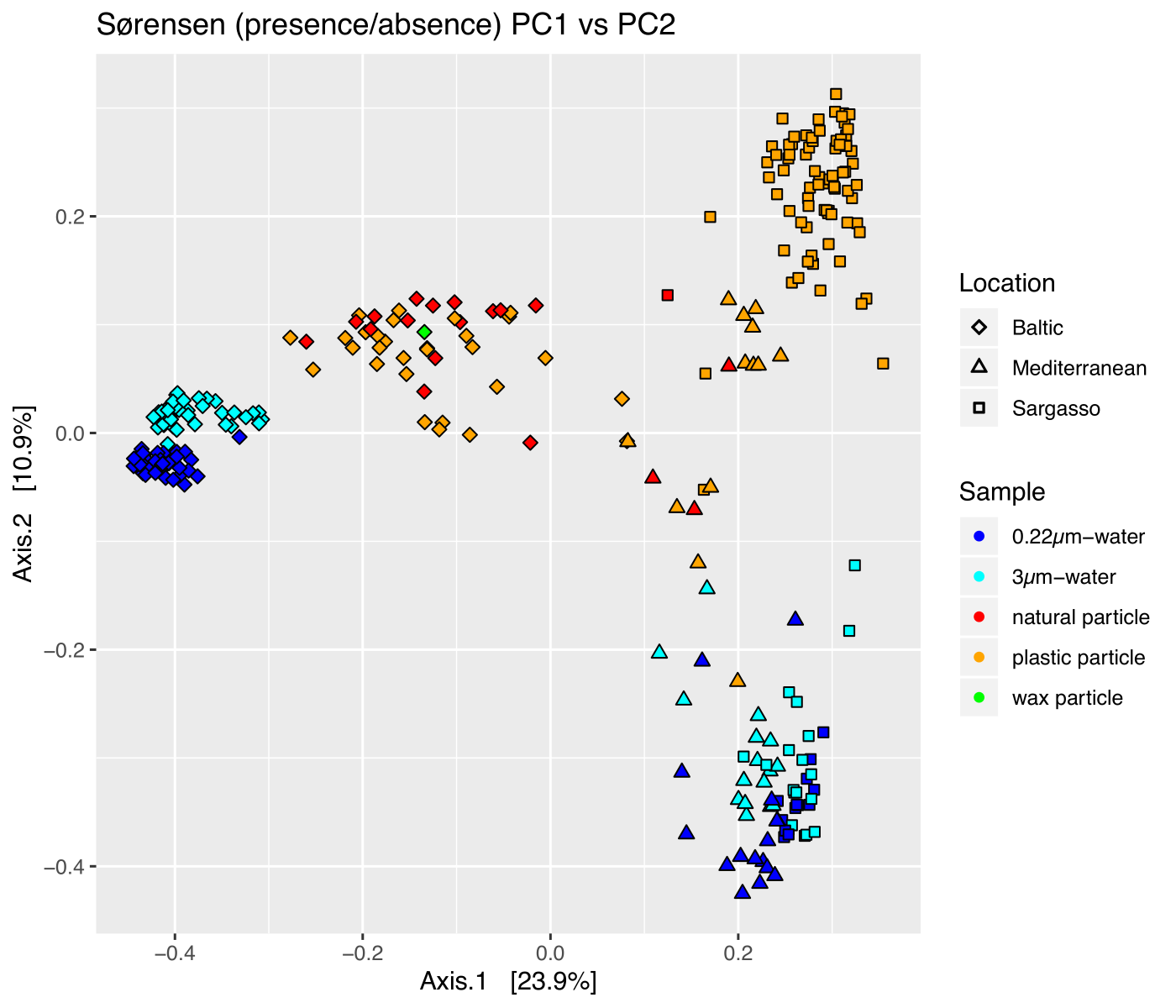

Supplement: FIG S3 [file msphere.00851-20-sf003.tif]

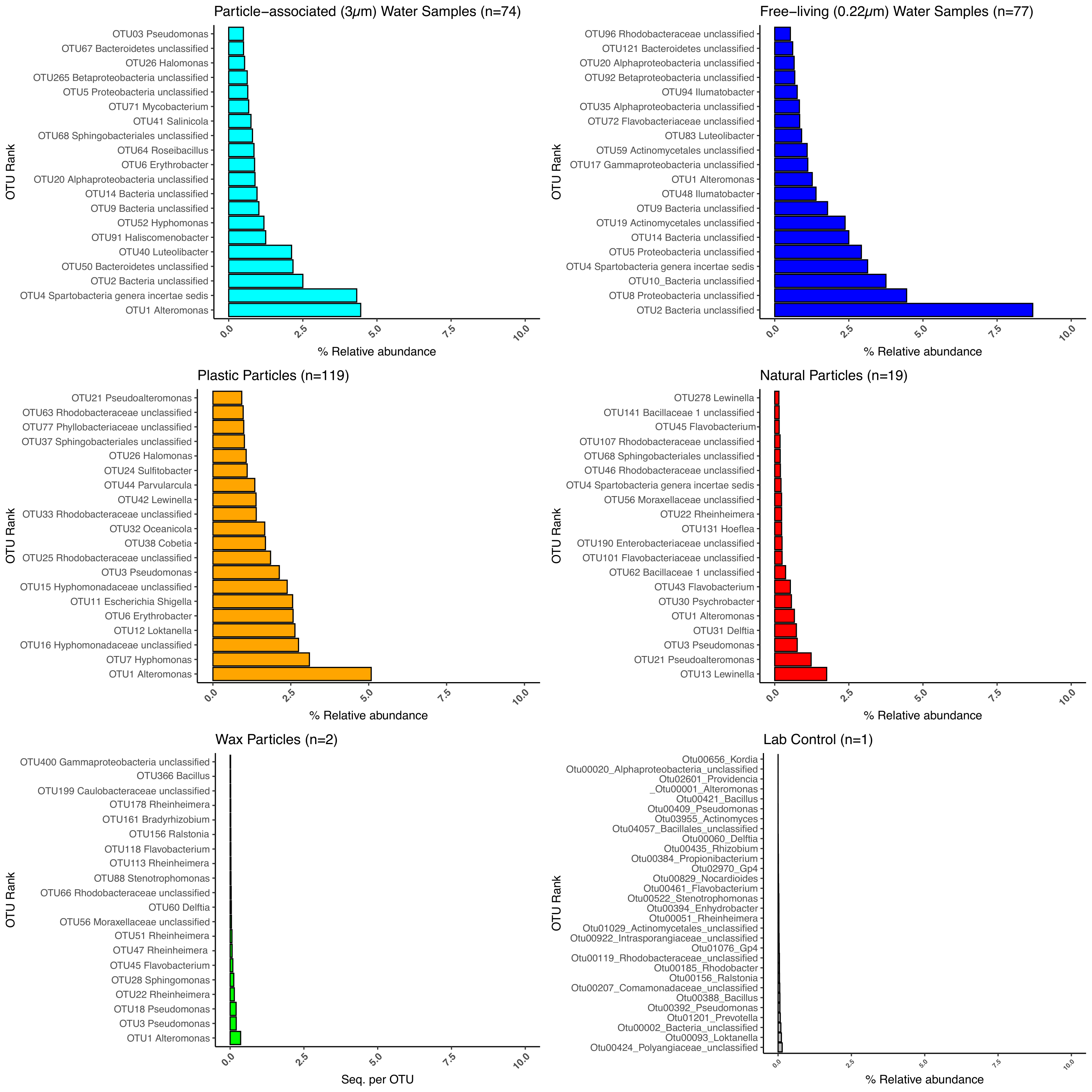

Supplement: FIG S4 [file msphere.00851-20-sf004.tif]

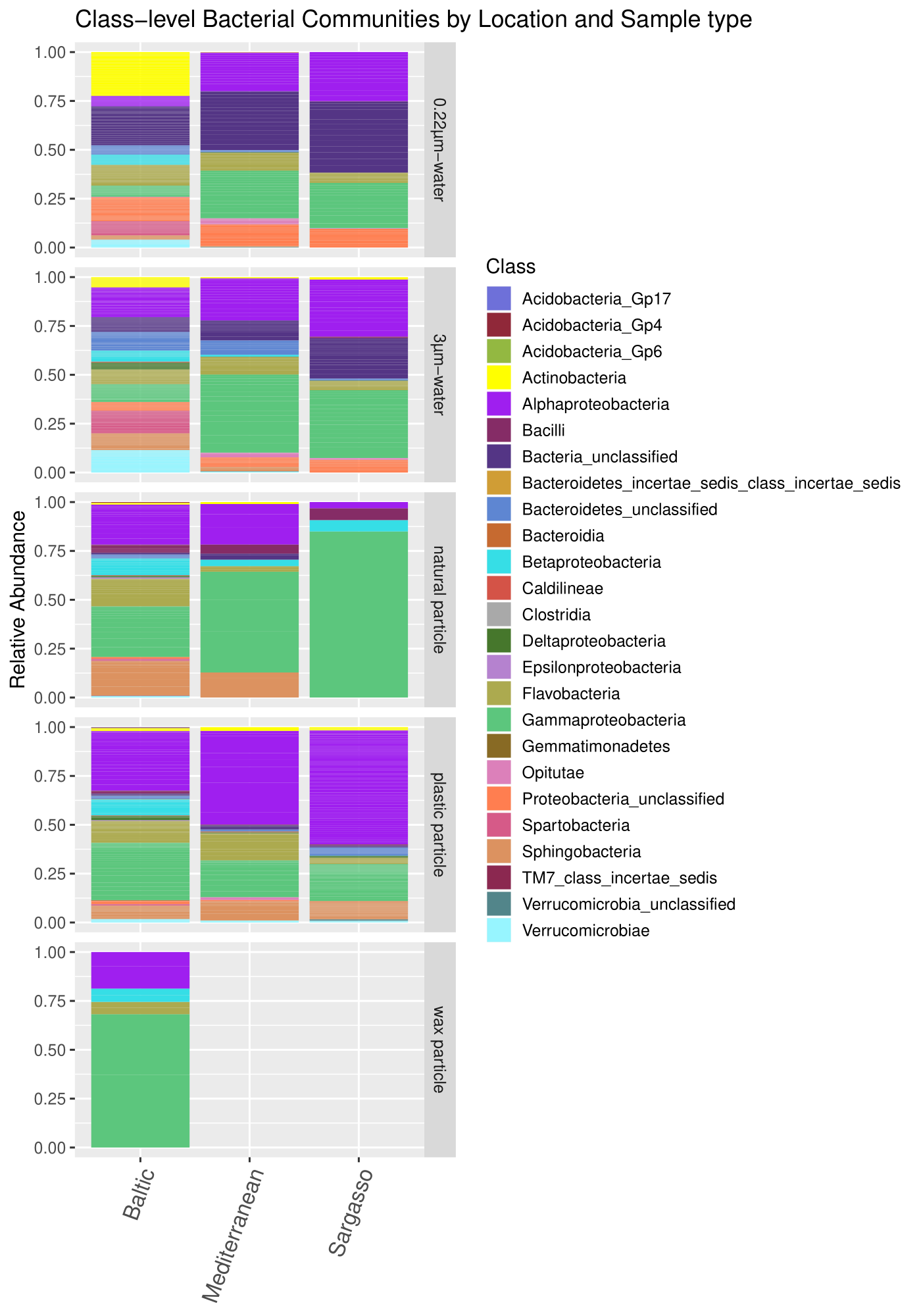

Supplement: FIG S5 [file msphere.00851-20-sf005.tif]

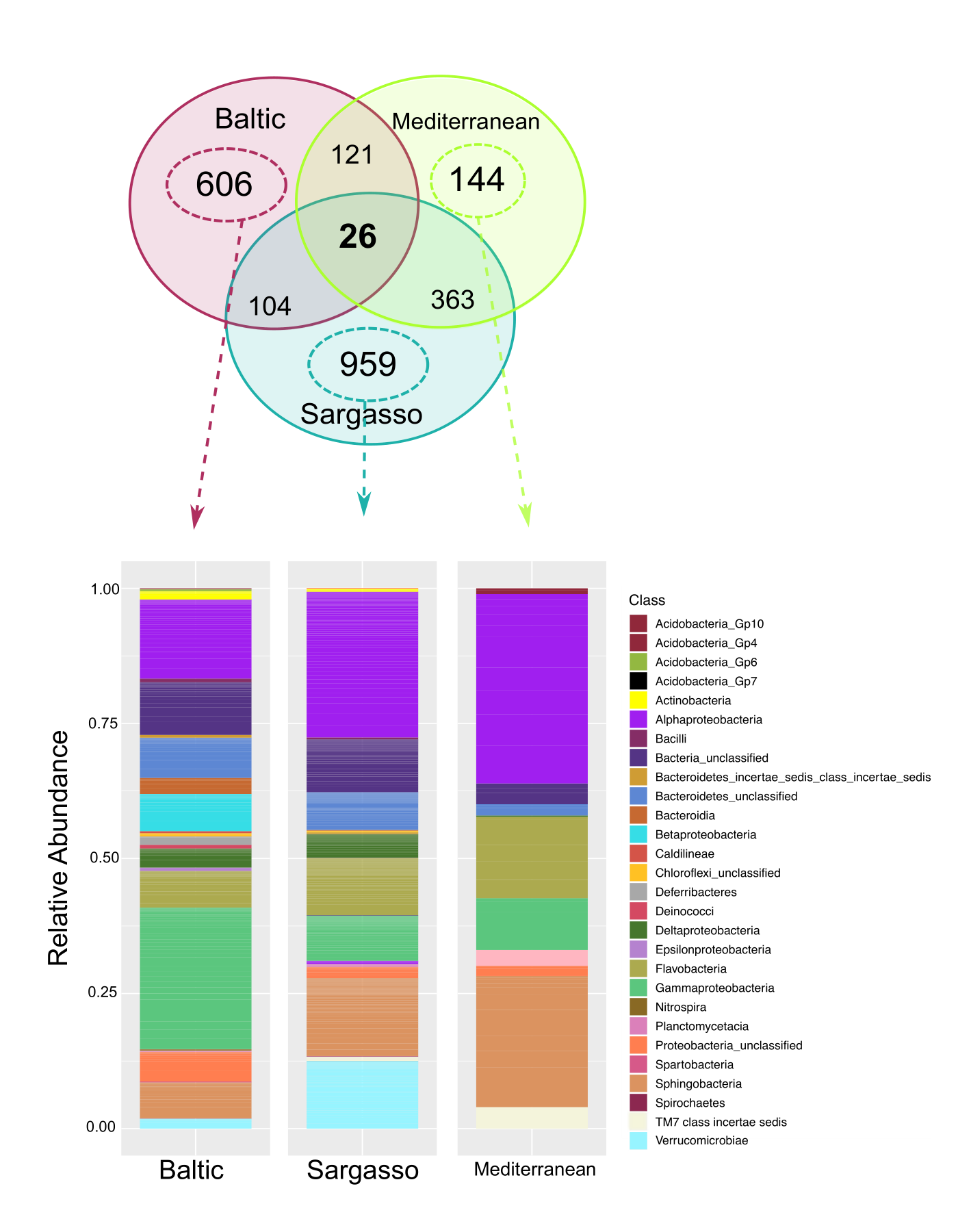

Supplement: FIG S6 [file msphere.00851-20-sf006.tif]

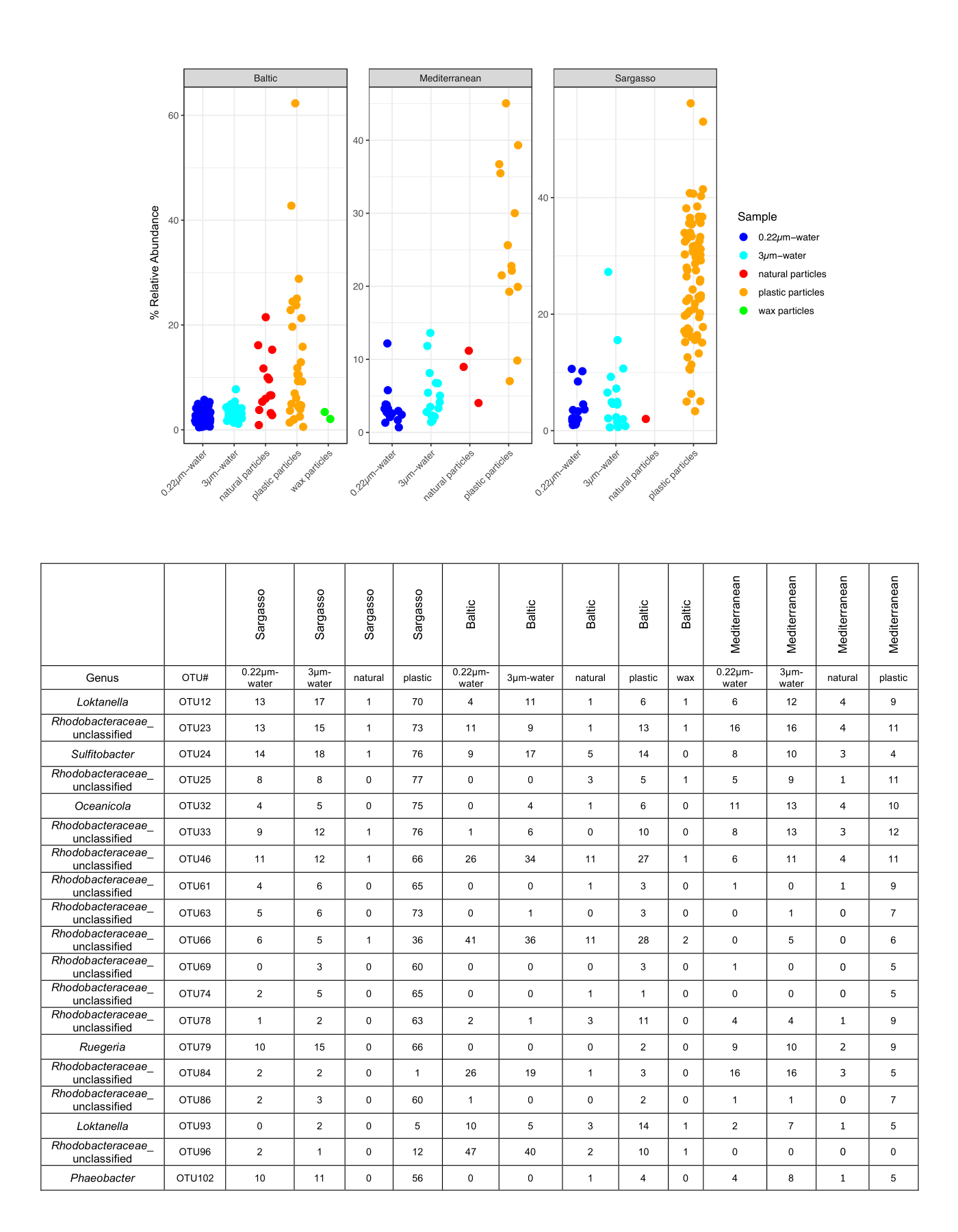

Supplement: FIG S7 [file msphere.00851-20-sf007.tif]

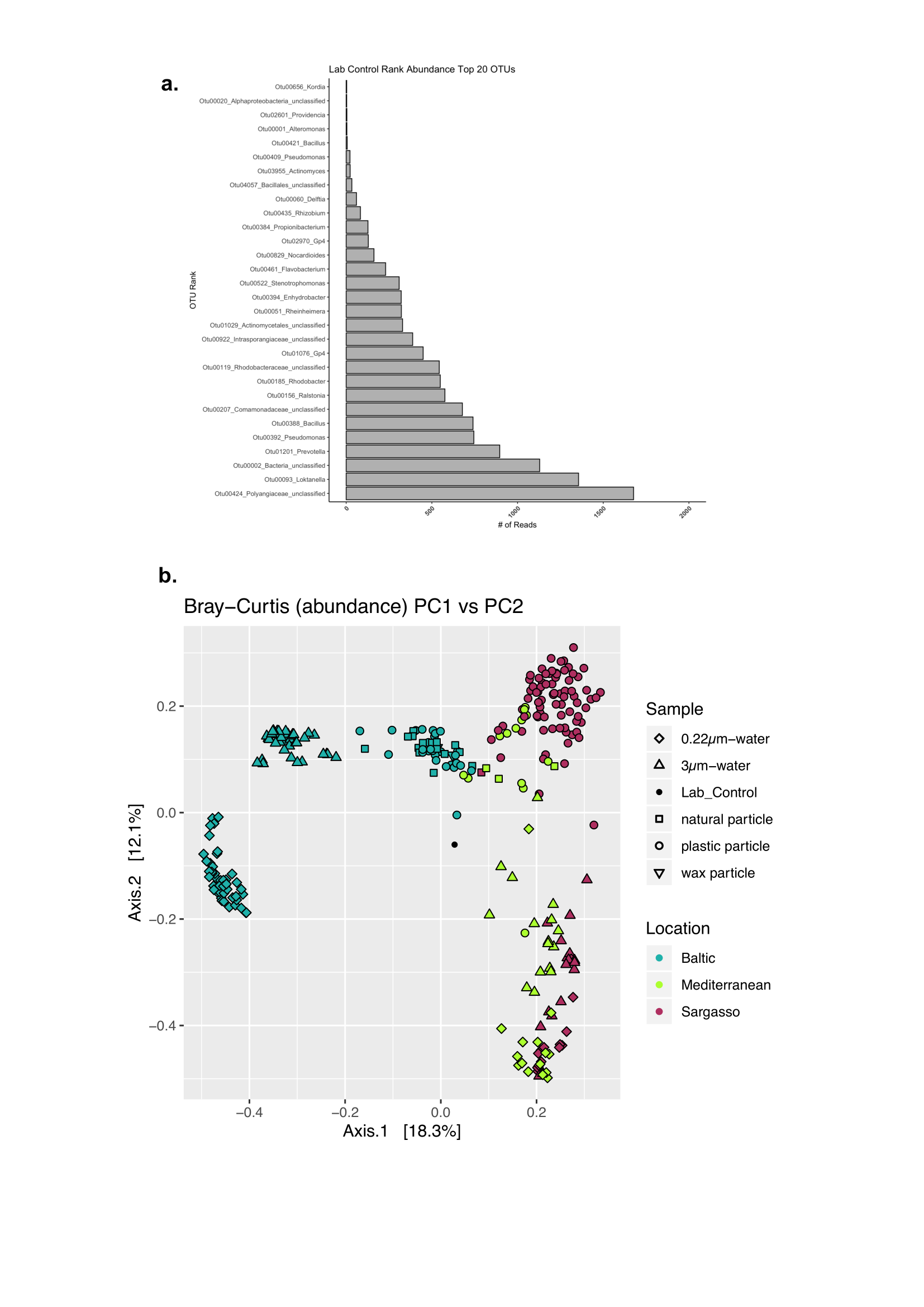

Supplement: FIG S8 [file msphere.00851-20-sf008.tif]
