# Supplementary material for: Cross-Hemisphere Study Reveals Geographically Ubiquitous, Plastic-Specific Bacteria Emerging from the Rare and Unexplored Biosphere
Source: mSphere. 2021 Jun 9;6(3):e00851-20. doi: 10.1128/mSphere.00851-20 (PMC8265672; doi:10.1128/mSphere.00851-20)
Supplement: TABLE S1 [file msphere.00851-20-st001.docx]

|  | | | | | | |
| --- | --- | --- | --- | --- | --- | --- |
| **Alpha-Diversity Statistics** | | | | | | |
| **Sample types** | | | | | | |
|  | **Comparison** | **Test Type** | **Chi-squared** | **df** | **p-value** |  |
| **Richness** | **Mean vs Sample** | **Kruskal-Wallis** | 80.543 | 4 | **2.2E-16 ***** |  |
| **Richness** | **Mean vs Sample** | **Posthoc Kruskal Dunn with Chi-squared** | 0.22µm-water | 3µm-water | natural | plastic |
|  | | 3µm-water | **0.00025 ***** | - | - | - |
|  |  | natural | **8.7e-05 ***** | **1.3E-11 ***** | - | - |
|  |  | plastic | 0.05977 | **2.0E-12 ***** | 0.02852 | - |
|  |  | wax | 0.68816 | 0.05504 | 1 | 1 |
| **Richness** | **SD vs Sample** | **Kruskal-Wallis** | 72.397 | 4 | **7.075E-15 ***** |  |
| **Richness** | **SD vs Sample** | **Posthoc Kruskal Dunn with Chi-squared** | 0.22µm-water | 3µm-water | natural | plastic |
|  | | 3µm-water | 1 | - | - | - |
|  |  | natural | **4.4E-10 ***** | **3.1E-07 ***** | - | - |
|  |  | plastic | **4.6E-09 ***** | **0.00012 ***** | **0.01661 *** | - |
|  |  | wax | 0.13867 | 0.3653 | 1 | 1 |
| **Evenness** | **Mean vs Sample** | **Kruskal-Wallis** | 12.088 | 4 | **0.01671 *** |  |
| **Evenness** | **Mean vs Sample** | **Posthoc Kruskal Dunn with Chi-squared** | 0.22µm-water | 3µm-water | natural | plastic |
|  | | 3µm-water | 1 | - | - | - |
|  |  | natural | 0.092 | 0.040 | - | - |
|  |  | plastic | 1 | 0.849 | 0.470 | - |
|  |  | wax | 1 | 1 | 1 | 1 |
| **Evenness** | **SD vs Sample** | **Kruskal-Wallis** | 25.974 | 4 | **3.203e-05 ***** |  |
| **Evenness** | **SD vs Sample** | **Posthoc Kruskal Dunn with Chi-squared** | 0.22µm-water | 3µm-water | natural | plastic |
|  | | 3µm-water | 0.3263 | - | - | - |
|  |  | natural | 0.03548 | **0.00021 ***** | - | - |
|  |  | plastic | 1 | **0.00197 **** | 0.27798 | - |
|  |  | wax | 1 | 0.49319 | 1 | 1 |
|  | | | | | | |
| **Plastic vs Location Broad** | | | | | | |
|  | **Comparison** | **Test Type** | **Chi-squared** | **df** | **p-value** |  |
| **Richness** | **Mean vs Sample** | **Kruskal-Wallis** | 0.48482 | 2 | 0.7847 |  |
| **Richness** | **Mean vs Sample** | **Posthoc Kruskal Dunn with Chi-squared** | Baltic | Mediterranean |  |  |
|  |  | Mediterranean | 1 | - |  |  |
|  |  | Sargasso | 1 | 1 |  |  |
| **Evenness** | **Mean vs Sample** | **Kruskal-Wallis** | 0.74453 | 2 | 0.6892 |  |
| **Evenness** | **Mean vs Sample** | **Posthoc Kruskal Dunn with Chi-squared** | Baltic | Mediterranean |  |  |
|  |  | Mediterranean | 1 | - |  |  |
|  |  | Sargasso | 1 | 1 |  |  |
|  | | | | | | |
| **3um vs Location Broad** | | | | | | |
|  | **Comparison** | **Test Type** | **Chi-squared** | **df** | **p-value** |  |
| **Richness** | **Mean vs Sample** | **Kruskal-Wallis** | 5.442 | 2 | 0.06581 |  |
| **Richness** | **Mean vs Sample** | **Posthoc Kruskal Dunn with Chi-squared** | Baltic | Mediterranean |  |  |
|  |  | Mediterranean | 0.31 | - |  |  |
|  |  | Sargasso | 0.11 | 1.00 |  |  |
| **Evenness** | **Mean vs Sample** | **Kruskal-Wallis** | 5.9262 | 2 | 0.05166 |  |
| **Evenness** | **Mean vs Sample** | **Posthoc Kruskal Dunn with Chi-squared** | Baltic | Mediterranean |  |  |
|  |  | Mediterranean | 1 | - |  |  |
|  |  | Sargasso | **0.046 *** | 0.707 |  |  |
|  | | | | | | |
| **0.22um vs Location Broad** | | | | | | |
|  | **Comparison** | **Test Type** | **Chi-squared** | **df** | **p-value** |  |
| **Richness** | **Mean vs Sample** | **Kruskal-Wallis** | 3.168 | 2 | 0.2051 |  |
| **Richness** | **Mean vs Sample** | **Posthoc Kruskal Dunn with Chi-squared** | Baltic | Mediterranean |  |  |
|  |  | Mediterranean | 0.29 | - |  |  |
|  |  | Sargasso | 0.94 | 1 |  |  |
| **Evenness** | **Mean vs Sample** | **Kruskal-Wallis** | 39.29 | 2 | **2.94E-09 ***** |  |
| **Evenness** | **Mean vs Sample** | **Posthoc Kruskal Dunn with Chi-squared** | Baltic | Mediterranean |  |  |
|  |  | Mediterranean | **1.7E-06 ***** | - |  |  |
|  |  | Sargasso | **3.3E-06 ***** | 1 |  |  |

_­_

| **Beta-Diversity Statistics** | | | | | | | | | | | | | | |
| --- | --- | --- | --- | --- | --- | --- | --- | --- | --- | --- | --- | --- | --- | --- |
| **Bray-Curtis Dissimilarity Metric** | | | | | | | | | | | | | | |
|  | **Adonis** |  |  |  |  |  | **betadisper** |  |  |  |  |  | |  |
|  | **DF** | **Sum of Sqs** | **Mean Sqs** | **F.Model** | **R2** | **P(>F)** | **DF** | **Sum Sqs** | **Mean Sq** | **F** | **Perm** | | **P(>F)** | |
| **Plastic - Natural -Wax - 3um - 0.22um** | 4 | 22.1 | 5.525 | 16.64 | 0.1795 | **0.001 ***** | 4 | 1.288 | 0.322 | 19.81 | 999 | | **0.001 ***** | |
| **Plastic - 3um** | 1 | 7.84 | 7.84 | 20.38 | 0.096 | **0.001 ***** | 1 | 0.064 | 0.064 | 10.04 | 999 | | **0.004 **^†^** | |
| **Plastic - 0.22um** | 1 | 14.66 | 14.66 | 43.3 | 0.1827 | **0.001 ***** | 1 | 1.037 | 1.037 | 43.5 | 999 | | **0.001 ***^†^** | |
| **3um - 0.22um** | 1 | 6.115 | 6.115 | 20.64 | 0.1217 | **0.001 ***** | 1 | 0.4525 | 0.45247 | 16.733 | 999 | | **0.001***** | |
| **Plastic - Natural** | 1 | 2.42 | 2.42 | 6.125 | 0.043 | **0.001***** | 1 | 0.0023 | 0.0023 | 0.319 | 999 | | 0.603**^†^** | |
| **Plastic - Wax** | 1 | 0.978 | 0.978 | 2.50 | 0.02 | **0.001***** | 1 | 0.315 | 0.315 | 42.48 | 999 | | **0.001 ***^†^** | |
|  | | | | | | | | | | | | | | |
|  | **Adonis** |  |  |  |  |  | **betadisper** |  |  |  |  |  | |  |
|  | **DF** | **Sum of Sqs** | **Mean Sqs** | **F.Model** | **R2** | **P(>F)** | **DF** | **Sum Sqs** | **Mean Sq** | **F** | **N.Perm** | | **P(>F)** | |
| **Baltic - Mediterranean - Sargasso** | 2 | 21.26 | 10.63 | 30.06 | 0.173 | **0.001***** | 2 | 0.0238 | 0.0119 | 0.917 | 999 | | 0.385 **^†^** | |
| **Baltic - Sargasso** | 1 | 16.84 | 16.84 | 48.95 | 0.169 | **0.001***** | 1 | 0.02 | 0.02 | 0.113 | 999 | | 0.222 | |
| **Baltic - Mediterranean** | 1 | 9.68 | 9.68 | 28.25 | 0.136 | **0.001***** | 1 | 0.0001 | 0.000062 | 0.0028 | 999 | | 0.964 **^†^** | |
| **Sargasso - Mediterranean** | 1 | 3.7 | 3.7 | 10.611 | 0.063 | **0.001***** | 1 | 0.096 | 0.00964 | 1.07 | 999 | | 0.305 **^†^** | |
|  | | | | | | | | | | | | | | |
|  | **Adonis** |  |  |  |  |  | **betadisper** |  |  |  |  | |  | |
| **Baltic**  **Samples** | DF | Sum of Sqs | Mean Sqs | F.Model | R2 | P(>F) | DF | Sum Sqs | Mean Sq | F | N.Perm | | P(>F) | |
| **Plastic - Natural - 3um - 0.22um** | 4 | 18.3 | 4.57 | 22.07 | 0.41 | **0.001***** | 4 | 3.069 | 0.767 | 128.6 | 999 | | **0.001***** | |
| **Plastic - 3um** | 1 | 4.56 | 4.56 | 16.5 | 0.2 | **0.001***** | 1 | 0.68 | 0.68 | 93.20 | 999 | | **0.001***** | |
| **Plastic - 0.22um** | 1 | 8.4 | 8.4 | 44.15 | 0.377 | **0.001***** | 1 | 2.49 | 2.49 | 684.1 | 999 | | **0.001***** | |
| **3um - 0.22um** | 1 | 8.18 | 8.18 | 67.35 | 0.442 | **0.001***** | 1 | 0.6478 | 0.6478 | 95.96 | 999 | | **0.001***** | |
| **Plastic - Natural** | 1 | 0.76 | 0.76 | 1.965 | 0.0457 | **0.001***** | 1 | 0.024 | 0.024 | 5.65 | 999 | | **0.012 *** | |
| **Plastic - Wax** | 1 | 0.62 | 0.62 | 1.585 | 0.0535 | **0.21 *** | 1 | 0.3144 | 0.3144 | 97.3 | 999 | | **0.001 ***^†^** | |
|  | | | | | | | | | | | | | | |
|  | **Adonis** |  |  |  |  |  | **betadisper** |  |  |  |  | |  | |
| **Sargasso Samples** | DF | Sum of Sqs | Mean Sqs | F.Model | R2 | P(>F) | DF | Sum Sqs | Mean Sq | F | N.Perm | | P(>F) | |
| **Plastic - Natural - 3um - 0.22um** | 3 | 10.21 | 3.404 | 12.967 | 0.266 | **0.001***** | 3 | 1.677 | 0.559 | 75.4 | 999 | | **0.001***** | |
| **Plastic - 3um** | 1 | 5.26 | 5.26 | 18.3 | 0.163 | **0.001***** | 1 | 0.417 | 0.417 | 88.04 | 999 | | **0.001***^†^** | |
| **Plastic - 0.22um** | 1 | 5.73 | 5.73 | 20.29 | 0.184 | **0.001***** | 1 | 1.156 | 1.156 | 139.08 | 999 | | **0.001***^†^** | |
| **3um - 0.22um** | 1 | 0.880 | 0.880 | 7.093 | 0.1912 | **0.003**** | 1 | 0.1564 | 0.1564 | 10.655 | 999 | | **0.005**** | |
| **Plastic - Natural** | 1 | 0.602 | 0.602 | 1.9 | 0.023 | **0.013*** | 1 | 0.305 | 0.305 | 65.9 | 999 | | **0.001***** | |
|  | | | | | | | | | | | | | | |
|  | **Adonis** |  |  |  |  |  | **betadisper** |  |  |  |  | |  | |
| **Mediterranean**  **Samples** | DF | Sum of Sqs | Mean Sqs | F.Model | R2 | P(>F) | DF | Sum Sqs | Mean Sq | F | N.Perm | | P(>F) | |
| **Plastic - Natural - 3um - 0.22um** | 3 | 5.023 | 1.675 | 6.6213 | 0.311 | **0.001***** | 3 | 0.753 | 0.25 | 14.19 | 999 | | **0.001***** | |
| **Plastic - 3um** | 1 | 1.832 | 1.832 | 5.845 | 0.178 | **0.001***** | 1 | 0.165 | 0.165 | 13.38 | 999 | | **0.003**** | |
| **Plastic - 0.22um** | 1 | 3.0 | 3.0 | 12.1 | 0.309 | **0.001***** | 1 | 0.718 | 0.718 | 37.45 | 999 | | **0.001***** | |
| **3um - 0.22um** | 1 | 1.758 | 1.758 | 9.86 | 0.247 | **0.001***** | 1 | 0.216 | 0.216 | 9.186 | 999 | | **0.005**** | |
| **Plastic - Natural** | 1 | 0.411 | 0.411 | 0.9968 | 0.066 | 0.437 | 1 | 0.0028 | 0.0028 | 0.50 | 999 | | 0.477 **^†^** | |
|  | | | | | | | | | | | | | | |
|  | **Adonis** |  |  |  |  |  | **betadisper** |  |  |  |  | |  | |
| **Plastic Samples** | DF | Sum of Sqs | Mean Sqs | F.Model | R2 | P(>F) | DF | Sum Sqs | Mean Sq | F | N.Perm | | P(>F) | |
| **Plastic types (all)** | 11 | 6.524 | 0.593 | 1.58 | 0.1386 | **0.001 ***** | 11 | 2.76 | 0.25 | 39.27 | 999 | | **0.001 ***** | |
| **PE vs PP** | 1 | 1 | 1 | 2.64 | 0.026 | **0.001 ***** | 1 | 0.024 | 0.024 | 3.59 | 999 | | 0.073 | |
| **Baltic - Mediterranean - Sargasso** | 2 | 6.097 | 3.05 | 8.54 | 0.13 | **0.001 ***** | 2 | 0.124 | 0.062 | 15.131 | 999 | | **0.001 ***^†^** | |
| **Baltic - Sargasso** | 1 | 5 | 5 | 14.25 | 0.12 | **0.001 ***** | 1 | 0.072 | 0.072 | 17.94 | 999 | | **0.001 ***** | |
| **Baltic - Mediterranean** | 1 | 1.61 | 1.61 | 3.92 | 0.91 | **0.001 ***** | 1 | 0.0001 | 0.0001 | 0.032 | 999 | | 0.865 | |
| **Sargasso - Mediterranean** | 1 | 1.46 | 1.46 | 4.4 | 0.048 | **0.001 ***** | 1 | 0.638 | 0.638 | 12.92 | 999 | | **0.001 ***** | |
|  |  |  |  |  |  |  |  |  |  |  |  | |  | |
|  | **Adonis** |  |  |  |  |  | **betadisper** |  |  |  |  | |  | |
| **Particle-associated Samples** | DF | Sum of Sqs | Mean Sqs | F.Model | R2 | P(>F) | DF | Sum Sqs | Mean Sq | F | N.Perm | | P(>F) | |
| **Baltic - Mediterranean - Sargasso** | 2 | 11.64 | 5.819 | 30.47 | 0.462 | **0.001 ***** | 2 | 0.0526 | 0.0263 | 2.525 | 999 | | 0.095 | |
| **Baltic - Sargasso** | 1 | 7.2088 | 7.2088 | 45.451 | 0.44801 | **0.001 ***** | 1 | 0.000049 | 0.00004934 | 0.0172 | 999 | | 0.902 | |
| **Baltic - Mediterranean** | 1 | 5.9947 | 5.9947 | 34.664 | 0.39096 | **0.001 ***** | 1 | 0.033258 | 0.033258 | 7.5213 | 999 | | **0.009 **** | |
| **Sargasso - Mediterranean** | 1 | 7.2088 | 7.2088 | 45.451 | 0.44801 | **0.001 ***** | 1 | 0.000865 | 0.00086511 | 0.2932 | 999 | | 0.578 | |
|  |  |  |  |  |  |  |  |  |  |  |  | |  | |
|  | **Adonis** |  |  |  |  |  | **betadisper** |  |  |  |  | |  | |
| **Free-living Samples** | DF | Sum of Sqs | Mean Sqs | F.Model | R2 | P(>F) | DF | Sum Sqs | Mean Sq | F | N.Perm | | P(>F) | |
| **Baltic - Mediterranean - Sargasso** | 2 | 13.1090 | 6.5545 | 83.058 | 0.69181 | **0.001 ***** | 2 | 0.05258 | 0.026289 | 2.5246 | 999 | | 0.095 | |
| **Baltic - Sargasso** | 1 | 7.0960 | 7.0960 | 67.86 | 0.53492 | **0.001 ***** | 1 | 0.012122 | 0.0121218 | 7.5942 | 999 | | **0.014 *** | |
| **Baltic - Mediterranean** | 1 | 7.0845 | 7.0845 | 58.688 | 0.49034 | **0.001 ***** | 1 | 0.13836 | 0.138357 | 52.114 | 999 | | **0.001 ***** | |
| **Sargasso - Mediterranean** | 1 | 7.0960 | 7.0960 | 67.86 | 0.53492 | **0.001 ***** | 1 | 0.020019 | 0.0200190 | 12.005 | 999 | | **0.003 **** | |
| Significant codes: 0 ‘*******’ 0.001 ‘******’ 0.01 ‘***’** 0.05, ^†^ symbol indicates when correction for unequal group sizes was applied to the test | | | | | | | | | |  |  | |  | |
|  | | | | | | | | | | | | | | |
